# HLA A*24:02–restricted T cell receptors cross-recognize bacterial and preproinsulin peptides in type 1 diabetes

**DOI:** 10.1172/JCI164535

**Published:** 2024-09-17

**Authors:** Garry Dolton, Anna Bulek, Aaron Wall, Hannah Thomas, Jade R. Hopkins, Cristina Rius, Sarah A.E. Galloway, Thomas Whalley, Li Rong Tan, Théo Morin, Nader Omidvar, Anna Fuller, Katie Topley, Md Samiul Hasan, Shikha Jain, Nirupa D’Souza, Thomas Hodges-Hoyland, the TIRID Consortium, Owen B. Spiller, Deborah Kronenberg-Versteeg, Barbara Szomolay, Hugo A. van den Berg, Lucy C. Jones, Mark Peakman, David K. Cole, Pierre J. Rizkallah, Andrew K. Sewell

**Affiliations:** 1 Division of Infection and Immunity, Cardiff University School of Medicine, Cardiff, Wales, United Kingdom.; 2 Cwm Taf Morgannwg University Health Board, Princess of Wales Hospital, Mountain Ash, United Kingdom.; 3 Ashgrove Surgery, Pontypridd, United Kingdom.; 4 The TIRID Consortium is detailed in Supplemental Acknowledgments.; 5 Department of Immunobiology, King’s College London, United Kingdom.; 6 Systems Immunology Research Institute, Cardiff University, Cardiff, United Kingdom.; 7 Warwick Systems Biology Centre, University of Warwick, Coventry, United Kingdom.

**Keywords:** Autoimmunity, Immunology, Diabetes, Structural biology, T cell receptor

## Abstract

CD8^+^ T cells destroy insulin-producing pancreatic β cells in type 1 diabetes through HLA class I–restricted presentation of self-antigens. Combinatorial peptide library screening was used to produce a preferred peptide recognition landscape for a patient-derived T cell receptor (TCR) that recognized the preproinsulin-derived (PPI-derived) peptide sequence LWMRLLPLL in the context of disease risk allele HLA A*24:02. Data were used to generate a strong superagonist peptide, enabling production of an autoimmune HLA A*24:02–peptide–TCR structure by crystal seeding. TCR binding to the PPI epitope was strongly focused on peptide residues Arg4 and Leu5, with more flexibility at other positions, allowing the TCR to strongly engage many peptides derived from pathogenic bacteria. We confirmed an epitope from *Klebsiella* that was recognized by PPI-reactive T cells from 3 of 3 HLA A*24:02^+^ patients. Remarkably, the same epitope selected T cells from 7 of 8 HLA A*24^+^ healthy donors that cross-reacted with PPI, leading to recognition and killing of HLA A*24:02^+^ cells expressing PPI. These data provide a mechanism by which molecular mimicry between pathogen and self-antigens could have resulted in the breaking of self-tolerance to initiate disease.

## Abstract 2

Bacterial peptides can generate insulin-specific killer T-cells, providing a molecular mechanism in breaking of self-tolerance to initiate type 1 diabetes.<br>

## Graphical abstract

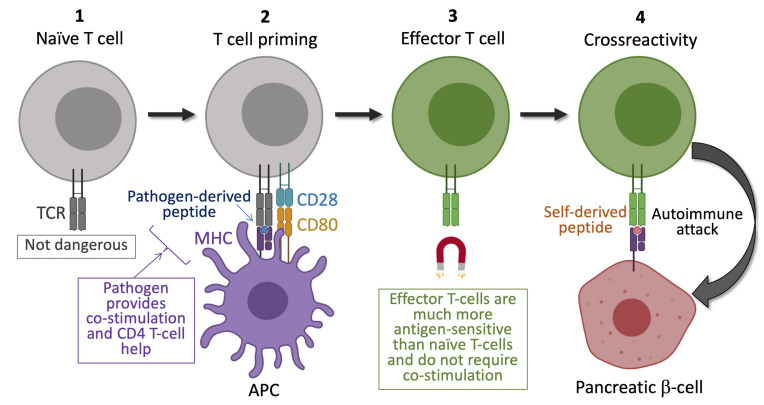

## Introduction

Multiple lines of evidence show that type 1 diabetes (T1D) develops due to CD8^+^ T cell–mediated destruction of insulin-producing pancreatic β cells. Human leukocyte antigen class I (HLA-I), the primary antigen presentation platform for CD8^+^ T cells, is strongly implicated in disease onset and progression. Islets of Langerhans, which normally express very low levels of HLA-I (1), hyperexpress these CD8^+^ T cell recognition platforms in patients with T1D (2–4), and abrogation of major histocompatibility complex (MHC) class I expression in NOD mice confers disease protection (5, 6). Additionally, a detailed search involving more than 2,000 patients with T1D identified HLA B*39, HLA B*18, and HLA A*24 as being associated with T1D susceptibility, conferring a relative risk comparable to, or greater than, the more classically associated T1D genes, such as *PTPN22* (7). Subsequent studies at 4-digit resolution show that HLA B*39:06, HLA A*24:02, HLA A*02:01, HLA B*18:01, and HLA C*05:01 predispose toward disease (8). HLA A*02:01 and HLA B*39:06 have both been observed to mediate T1D in mouse models (9, 10). Other evidence, in addition to the disease linkage with HLA-I, implicates CD8^+^ T cells in disease initiation, as they are the most abundant cell type in the leukocyte infiltrate during inflammation of islets early in disease, and there is an inverse correlation between the frequency of CD8^+^ T cell infiltrate and islet insulin expression (11). This insulitis-associated infiltrate is enriched in CD8^+^ T cells that respond to putative diabetogenic epitopes (12), with as many as 10% of CD8^+^ T cells in the pancreas being specific for a single preproinsulin (PPI) epitope during recent-onset disease (13). Importantly, PPI-specific T cells have been shown to attack and kill HLA-matched human β cells isolated from deceased organ donor samples (14, 15).

Here, we focus on HLA A*24:02, which has a strong T1D disease-predisposing effect, is associated with a younger age of disease onset, and presents a peptide of the sequence LWMRLLPLL comprising residues 3–11 of PPI on targets expressing the *INS* gene, including β cells (7, 14). PPI is considered to be a critical autoantigenic driver in the development of T1D and autoimmune diabetes in relevant preclinical models (16, 17). Replacement of the *INS1* gene in NOD mice with the human ortholog protects against disease, suggesting that key disease-causing PPI-derived T cell epitopes may differ in the 2 species (18). HLA A*24:02–PPI_3–11_–specific CD8^+^ T cells are expanded in patients with recent-onset T1D (14). We studied the well-characterized T1D patient–derived HLA A*24:02–PPI_3–11_–specific T cell clone 4C6, which has been shown to kill HLA A*24:02^+^ human β cells (14). Five T cell clones were isolated by single-cell sorting with HLA A*24:02–PPI_3–11_ tetramer from the blood of a patient with T1D taken at 2 time points 6 months apart; and, following expansion and sequencing, all 5 T cell clones were shown to express the 4C6 T cell receptor (TCR), which uses the TRAV5*01, TRAJ37*01 TCR α-chain, CD3α sequence CAEPSGNTGKLIF, paired with the TRB7-9*03, TRBJ2-7*01 β-chain, CD3β sequence CASSLHHEQYF (Supplemental Figure 1; supplemental material available online with this article; https://doi.org/10.1172/JCI164535DS1). No other TCR was identified, suggesting that the 4C6 T cell dominated the patient response to PPI_3–11_. We therefore undertook a detailed study of the peptide recognition landscape of the 4C6 TCR using a combinatorial peptide library (CPL) and generated a superagonist peptide ligand for this TCR that bound with a dissociation constant (*K_D_*) of 5.4 μM, more than 20-fold higher than that of the PPI-derived natural sequence. This superagonist peptide allowed us to crystallize the 4C6 TCR with a cognate ligand and then seed crystals with the natural PPI_3–11_ ligand to generate what we believe to be the first atomic-resolution structure of an HLA A*24:02–restricted TCR in complex with autoantigen. We further discovered pathogen-derived peptides that were thousands of times more potent at activating the 4C6 T cell than the PPI_3–11_ sequence. CD8^+^ T cells were pulled from the PBMCs of 7 of 8 HLA A*24^+^ healthy donors using the *Klebsiella*-derived peptide SLPRLFPLL. Remarkably, these T cells also stained with HLA A*24:02–PPI_3–11_ multimers and killed HLA A*24:02^+^ cells expressing PPI to provide a potential mechanism by which such T cells might become activated against self via molecular mimicry.

## Results

### 4C6 T cells exhibit a restricted pattern of recognition at peptide positions 4 and 5.

**Figure 1:**
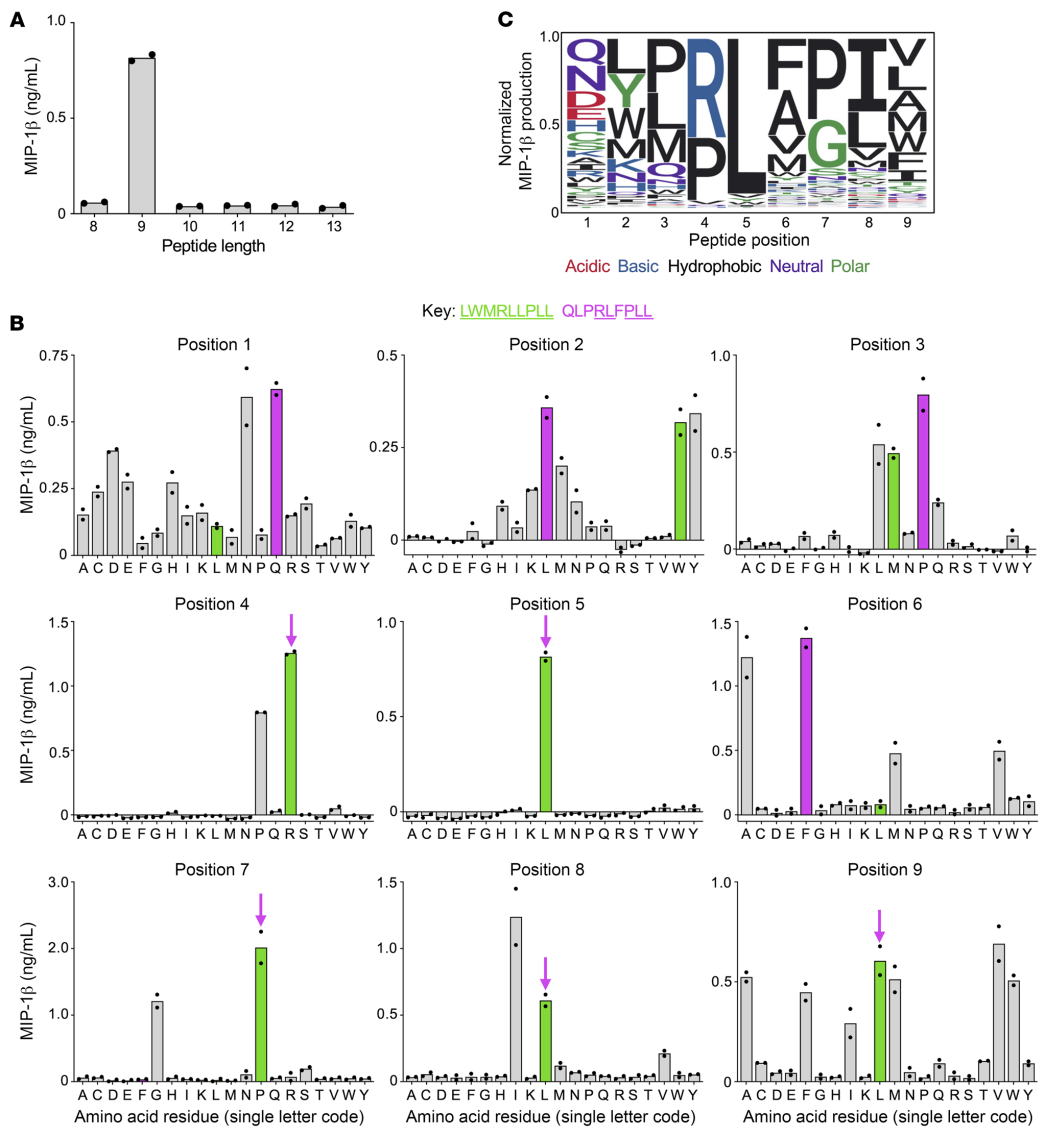
Sizing scan and positional scanning CPL screening of 4C6 T cells. (**A**) The 4C6 T cell clone was incubated overnight with sizing scan mixtures of defined amino acid length (*x* axis) using C1R-HLA A*24:02 as antigen-presenting cells. Assay supernatants used for MIP-1β ELISA. Data points shown for duplicate conditions. (**B**) Based on the results of the sizing scan, a 9-mer positional scanning CPL (PS-CPL) screen was performed, using 4C6 T cells and antigen-presenting cells and ELISA as in **A**. Key: Green bars indicate amino acid present in the natural PPI epitope; and magenta bars and arrows show amino acid present in the superagonist peptide (shared amino acid residues are underlined). Data points shown for duplicate conditions. A replicate assay gave similar results. (**C**) Motif logo plot summarizing the amino acid preference of 4C6 at each position of the PS-CPL.

We have previously determined that although CD8^+^ T cells can recognize vast numbers of individual peptide sequences (19, 20), HLA-I–restricted TCRs exhibit a preference for a specific length of peptide (21). A peptide sizing scan (21) showed that the 4C6 T cell exhibited a strong preference for peptides of 9 amino acids in length in the context of HLA A*24:02 (Figure 1A). A 9-mer CPL screen of the 4C6 T cell revealed that it exhibited constrained recognition across the center of the peptide (positions 4–8), with more flexibility at peptide positions 1–3 and 9 (Figure 1, B and C). These data indicated that the “index” amino acid found in the insulin-derived peptide (indicated by the green bars in Figure 1B) might be suboptimal in some peptide positions. This effect was particularly striking at position 6, where the phenylalanine sublibrary was vastly more potent than the one containing the index leucine residue. Specificity was most restricted at position 5, where only the leucine sublibrary was recognized. Overall, these data suggest that the 4C6 T cell can recognize a large number of peptides, with some being far more potent than the natural index insulin sequence.

### Generation of a potent superagonist peptide for the 4C6 T cell.

**Figure 2:**
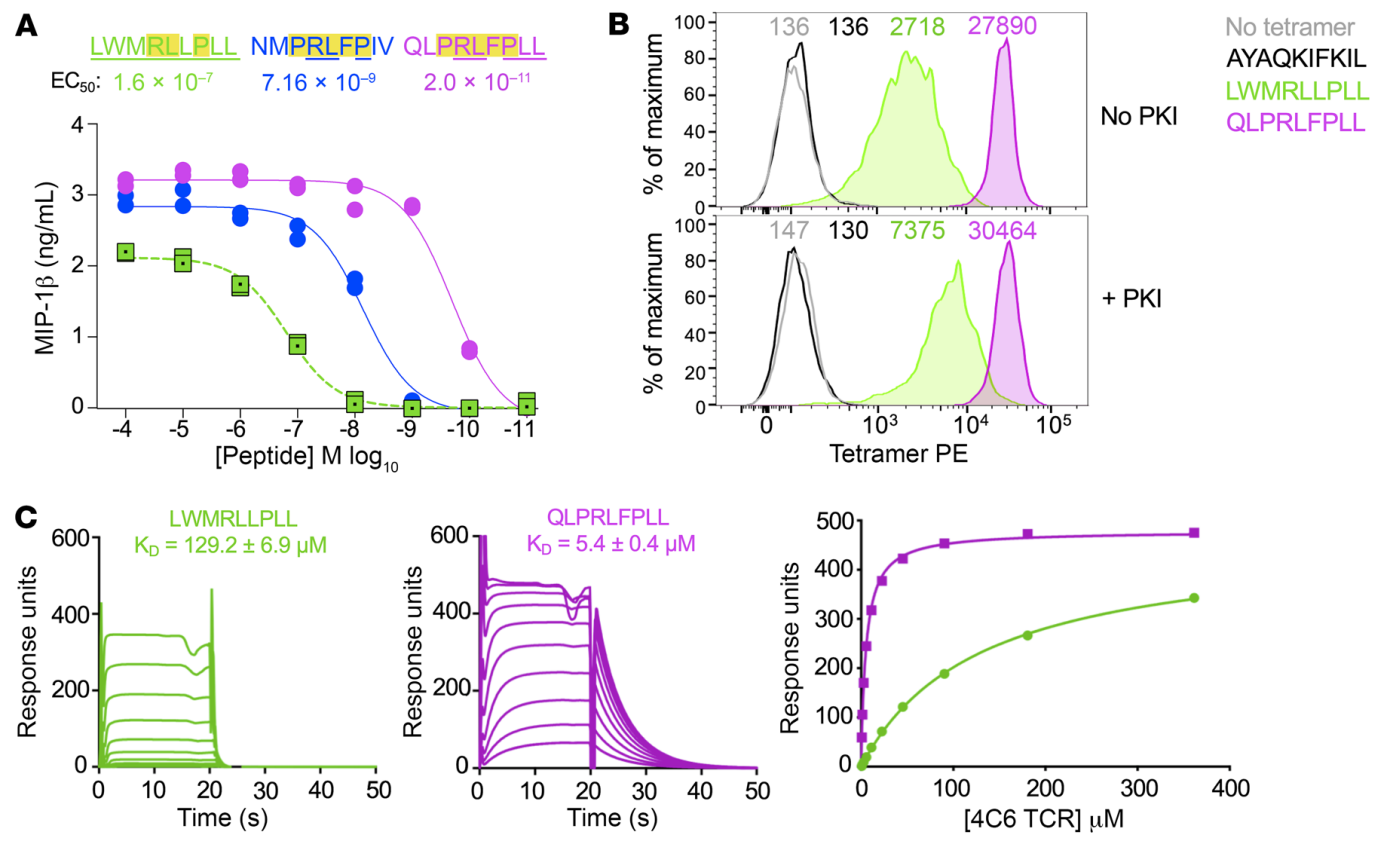
Cellular and biophysical analyses of the PPI and superagonist peptides. (**A**) Sensitivity of 4C6 T cell clone to PPI (LWMRLLPLL) and superagonists (NMPRLFPIV and QLPRLFPLL) peptides in a titration assay. Incubation overnight with C1R–HLA A*24:02 as antigen-presenting cells. Assay supernatants used for MIP-1β ELISA. Data points shown for duplicate conditions. Replicate assay including all candidate superagonist peptides tested for 4C6 as shown in Supplemental Figure 2. Underlined amino acid residues are the same as for the PPI peptide. Highlighted residues were present in both superagonist peptides. The most potent superagonist, QLPRLFPLL, was used for downstream experiments. (**B**) Staining of the 4C6 T cell clone with irrelevant (AYAQKIFKIL from CMV), PPI, and superagonist PE-conjugated tetramers. Tetramer used alone or following pretreatment with the protein kinase inhibitor (PKI) dasatinib. Mean fluorescence intensity of staining is displayed. Stained for CD8 APC-Vio770 and the viability stain VIVID. (**C**) SPR analysis of 4C6 TCR recognition of LWMRLLPLL (green) and QLPRLFPLL (magenta). SPR response to 10 serial dilutions of 4C6 was measured (left and center). *K_D_* values were calculated using nonlinear fit curve (*y* = [*P*1 × *x*]/[*P*2 + *x*]) (right).

CD8^+^ T cells are capable of recognizing millions of different individual peptides (20). This makes it unlikely that the index peptide will be optimal, and we have been able to find a more potent activating ligand for every HLA-I–restricted TCR for which we have attempted this approach, including public antiviral TCRs that respond to immunodominant epitopes (20, 22). The raw data shown in Figure 1B were analyzed using the algorithm developed by Szomolay et al. (23) to predict optimal ligands for the 4C6 T cell. The top 10 predicted ligands from a set of 500 peptides — sampled at random from the entire peptide universe with a bias toward good agonists using CPL data — were tested in titration assays, with NMPRLFPIV and QLPRLFPLL being the most potent at activating 4C6 (Supplemental Figure 2 and Figure 2A). The QLPRLFPLL sequence proved to be the best agonist, with an EC_50_ almost 10,000 times lower than the index PPI_3–11_ sequence as a crude peptide (Figure 2A). The QLPRLFPLL sequence is indicated by magenta bars and arrows in Figure 1B.

### The 4C6 T cell receptor binds to HLA A*24:02–PPI_3–11_ with relatively high affinity for an autoantigen.

The 4C6 T cell clone was successfully stained with HLA A*24:02–PPI_3–11_ tetramers (14) without the need for the protein kinase inhibitor dasatinib or other “tricks” usually required for staining of autoimmune T cells (24–26). The fact that the 4C6 T cell is amenable to regular peptide-HLA (pHLA) tetramer staining suggested that the 4C6 TCR might bind to HLA A*24:02–PPI_3–11_ with a relatively high affinity for an autoimmune TCR-pMHC interaction (25, 27). We confirmed that the 4C6 T cell could be stained by regular pHLA tetramer staining with HLA A*24:02–PPI_3–11_ reagents and showed that staining was more intense when the QLPRLFPLL superagonist sequence was used (Figure 2B). Soluble 4C6 TCR was produced as described previously (28) and shown to bind to HLA A*24:02–PPI_3–11_ with a *K_D_* of 129.2 μM by surface plasmon resonance (SPR). (Figure 2C). The affinity of the interaction between the 4C6 TCR and the QLPRLFPLL superagonist peptide (*K_D_* 5.4 μM) was more than 20-fold higher than that of the interaction between the 4C6 TCR and the PPI-derived LWMRLLPLL peptide, explaining why this ligand was almost 10,000-fold more potent in peptide titration assays (Figure 2A) and bound better as a pHLA multimer (Figure 2B).

### PPI_3-11_ residues Arg4 and Leu5 form a “peg-in-hole” network of bonding interactions with the 4C6 TCR.

**Figure 3:**
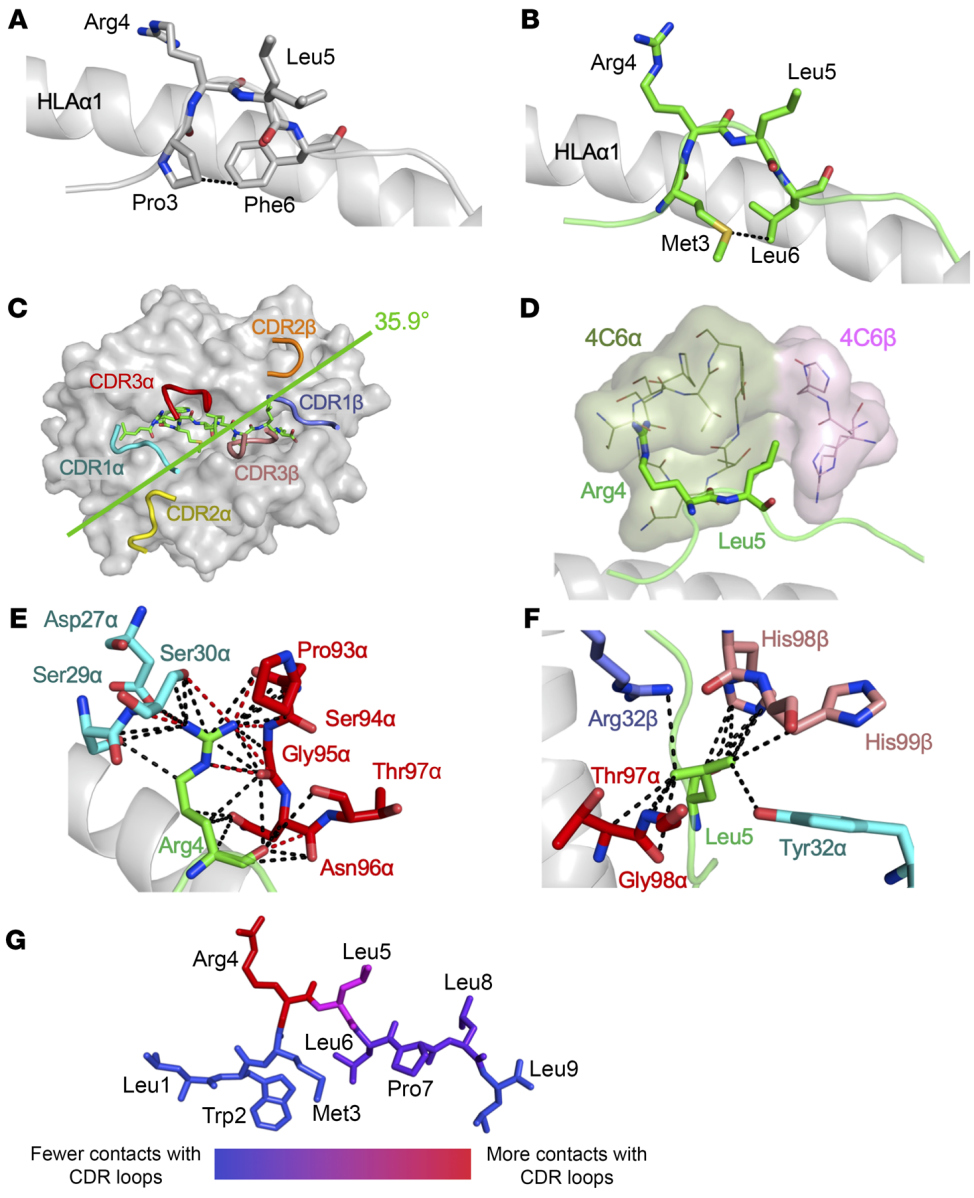
Structural analysis of the 4C6 TCR with superagonist and PPI peptides. (**A**) Structure of HLA A*24:02–QLPRLFPLL. Peptide shown as gray sticks, with MHC α-helix (gray) shown for orientation. Dotted lines represent VdW interactions. (**B**) Structure of 4C6-HLA A*24:02–LWMRLLPLL. Peptide shown as green sticks, with MHC α-helix (gray) shown for orientation. Dotted lines represent VdW interactions. (**C**) Top-down view of 4C6 TCR “footprint” on HLA A*24:02–LWMRLLPLL. 4C6 CDR loops shown as colored cartoon, with the peptide shown as green sticks. Green line and number indicate crossing angle. (**D**) Close-up of 4C6:HLA A24:02–LWMRLLPLL structure focusing on residues Arg4 and Leu5 (green sticks), which form a peg-in-hole formation inside the 4C6 TCR (lines and surface). (**E** and **F**) LWMRLLPLL peptide residues Arg4 (**E**) and Leu5 (**F**) shown as green sticks. Important 4C6 TCR residues are labeled. Black dotted lines indicate VdW interactions. Red dotted lines indicate hydrogen bonds. (**G**) Heatmap of 4C6 TCR contacts with the LWMRLLPLL peptide.

Attempts to generate crystals of HLA A*24:02 bound to PPI_3–11_ failed, but we were able to generate crystals of HLA A*24:02 bound to the QLPRLFPLL superagonist peptide that diffracted at 2.2 Å using our optimized crystallization screen (Supplemental Figure 3A and Supplemental Table 1) (29). This structure showed that QLPRLFPLL bulged in the middle, with Arg4 and Leu5 pointing outward as likely TCR contact residues (Figure 3A). The pronounced bulge was probably caused by a combination of an intrapeptide Van der Waals (VdW) interaction between Pro3 and Phe6, likely explaining the prominence of phenylalanine at position 6 in the CPL screen, and interactions between the peptide and the HLA A*24:02 binding groove.

Multiple attempts to generate cocrystals of the 4C6 TCR in complex with HLA A*24:02–PPI_3–11_ failed. However, we were able to generate crystals of the 4C6 TCR in complex with the HLA A*24:02–QLPRLFPLL superagonist peptide. These crystals were then used to seed complexes with HLA A*24:02–PPI_3–11_, which diffracted at 2.48 Å to produce a high-resolution structure. Analysis of the LWMRLLPLL peptide in the 4C6–HLA A*24:02–PPI_3–11_ complex demonstrated a conformation virtually identical to that of the QLPRLFPLL superagonist peptide, with the peptide bulged to project peptide residues 4 and 5 out of the HLA A*24:02 peptide-binding groove. A VdW interaction between the side-chains of the LWMRLLPLL Met3 and Leu6 residues likely helped support the central bulge in a manner analogous to that observed in the HLA A*24:02–QLPRLFPLL structure (Figure 3B and Supplemental Figure 3B). The 4C6-HLA A*24:02–PPI_3–11_ binding mode positioned the CDR1α, CDR3α, CDR1β, and CDR3β loops around the peptide bulge, while the CDR2α and CDR2β loops were largely positioned over the HLA A*24:02 molecule surface (Figure 3C). The positioning of the CDR loops enabled the 4C6 TCR to make substantial contacts with the solvent exposed residues in both peptides, with the side-chains of Arg4 and Leu5 forming a tight “peg-in-hole”–like interaction with a pocket formed by the TCR CDR1α, CDR3α, CDR1β, and CDR3β loops (Figure 3, D–F). Interaction with Arg4 and Leu5 accounted for 72% of contacts (51% and 21% respectively) between the LWMRLLPLL peptide and the TCR CDR loops (represented as a heatmap in Figure 3G). The dominance of contacts with this motif explained the restricted recognition at positions 4 and 5 in the CPL scan (Figure 1B). Arg4 made 34 contacts across the following residues: Asp27α, Ser29α, and Ser30α from CDR1α; and Pro93α, Ser94α, Gly95α, Asn96α, and Thr97α from CDR3α (Figure 3E and Supplemental Table 2). Leu5 made 14 VdW contacts across the following residues: Tyr32α from CDR1α; Thr97α and Gly98α from CDR3α; Arg32 from CDR1β; and His98 and His99 from CDR3β (Figure 3F and Supplemental Table 2).

Comparison of the 4C6 TCR in complex with both HLA A*24:02–QLPRLFPLL and HLA A*24:02–LWMRLLPLL revealed a virtually identical canonical binding mode, with the TCRα chain and TCRβ chain over the N- and C-termini of the peptide, respectively (Supplemental Figure 3, C–E). However, structural data suggest that despite binding over 20-fold more strongly to HLA A*24:02–QLPRLFPLL than HLA A*24:02–LWMRLLPLL, the 4C6 TCR made 15 fewer interactions with HLA A*24:02–QLPRLFPLL (Supplemental Tables 2 and 3). Several factors may have contributed to the increased affinity of the 4C6 TCR for HLA A*24:02–QLPRLFPLL compared with HLA A*24:02–LWMRLLPLL. First, increased VdW interactions between Pro3 and Phe6 within the superagonist peptide during 4C6 TCR binding may have contributed greater stabilization of the Arg4/Leu5 “peg” above the HLA A*24:02 binding platform (Supplemental Figure 3B and Supplemental Figure 4A). It was also evident that the superagonist peptide had a suboptimal MHC anchor at position 2 (Supplemental Figure 4B), a situation that we have previously observed in superagonist peptides (30, 31). Maintenance of the natural, and suboptimal, position 2 HLA A*02:01 anchor within the melanoma-associated Melan-A peptide EAAGIGILTV allows a cognate TCR to pull the peptide out of the MHC binding groove toward itself, thereby making a stronger interaction (32–34). Additionally, thermodynamic comparison of 4C6–HLA A*24:02–LWMRLLPLL and 4C6–HLA A*24:02–QLPRLFPLL showed that interaction with the PPI-derived sequence was enthalpically unfavorable and entropically favorable, whereas the reverse was true for the superagonist structure. These thermodynamics data indicate a net formation of interactions in the 4C6–HLA A*24:02–QLPRLFPLL complex, perhaps in part due to the suboptimal P2 anchor, which may contribute to the more than 20-fold-higher affinity (Supplemental Figure 4C).

### 4C6 T cells respond to a large number of different 9-mer peptides in the context of HLA A*24:02.

We next used the importance sampling technique we previously adapted for estimation of how many peptides an individual T cell could respond to (20). The raw data from the CPL screen shown in Figure 1B, with cysteine excluded, were normalized as previously described to provide a sampling distribution to bias the sample toward good agonists. A total of 60 peptides were drawn from an effective set of 3.16 × 10^6^ from the sampling entropy, as previously described (20). Of the 60 peptides, 8 were recognized with *p*EC_50_ greater than 5 (*p*EC_50_ is −1 × the base 10 logarithm [*p*] of the EC_50_, as used previously; ref. 20). Five were better agonists than the index peptide, suggesting that the 4C6 T cell would be capable of recognizing more than 1,000,000 different peptides as well as or better than the index PPI_3–11_ sequence used to kill human pancreatic β cells (Supplemental Figure 5). These results suggest that 4C6 T cells might be capable of strongly cross-reacting with a peptide sequence derived from a pathogen.

### Peptides-derived from human pathogens act as strong agonists of the 4C6 T cell.

**Figure 4:**
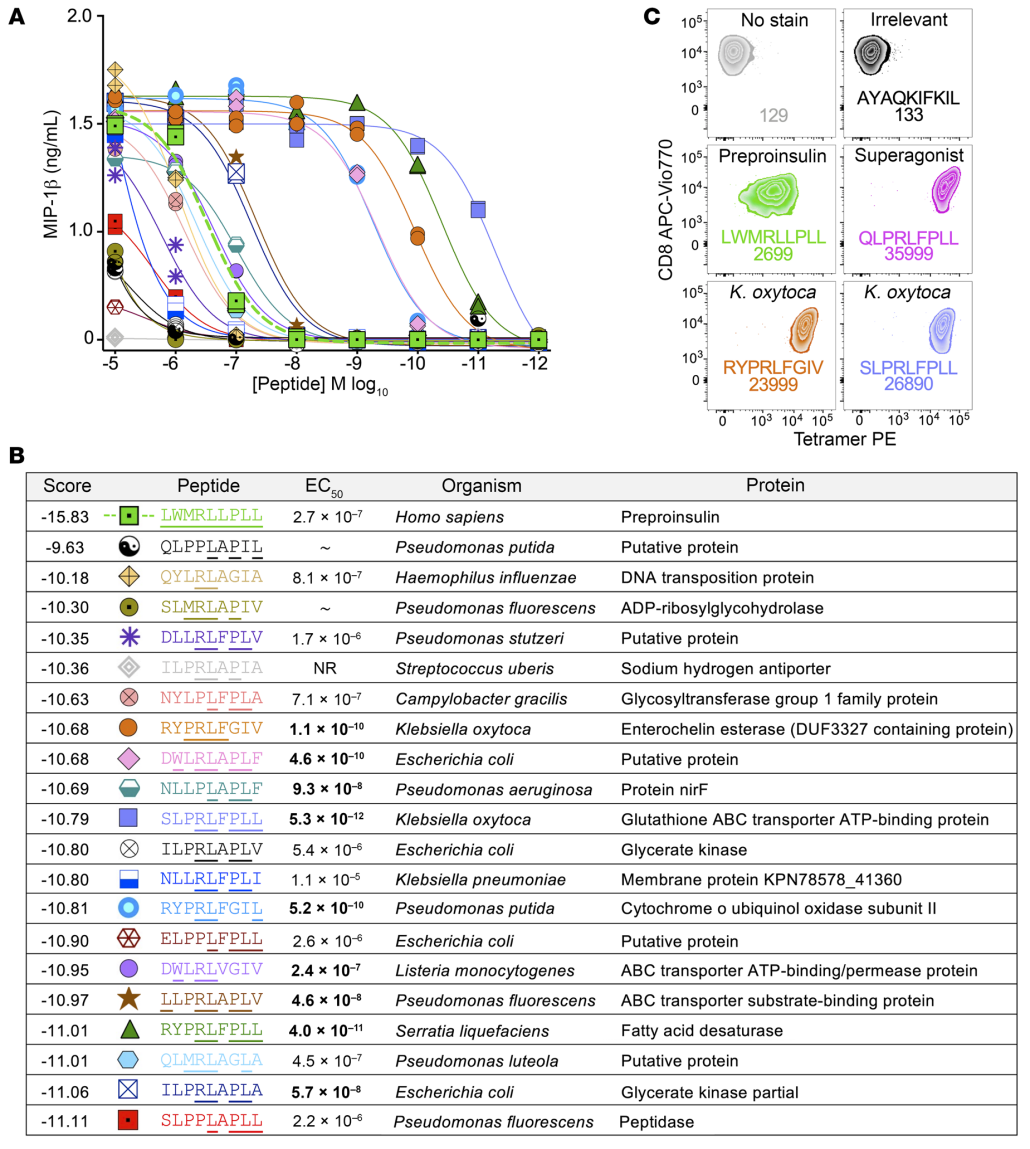
4C6 T cells cross-react with peptides derived from bacterial proteomes. PS-CPL data for 4C6 (Figure 1) was used to screen a database of infectious bacteria and the top 20 peptides selected for testing. (**A**) Peptide titrations using 4C6 with the top 20 bacteria peptides (listed in **B**). Incubation overnight with C1R–HLA A*24:02 as antigen-presenting cells. Assay supernatants used for MIP-1β ELISA. Data points shown for duplicate conditions. (**B**) Peptide sequence and origin. Scoring indicates prediction of how likely the peptide is to be recognized by 4C6 T cells, with the best-scoring peptide at the top. EC_50_ of activation in bold indicates peptides that acted as superagonists of the 4C6 T cell (ligand more potent than natural insulin sequence). One of the peptides gave no response (NR), and 2 of them gave ambiguous EC_50_ values (~) according to GraphPad Prism. (**C**) Staining of 4C6 T cell clone with pMHC tetramers bearing CMV irrelevant epitope (AYAQKIFKIL), PPI (LWMRLLPLL), superagonist (QLPRLFPLL), and *K*. *oxytoca* (RYPRLFGIV and SLPRLFPLL) PE tetramers. Staining performed without PKI. Mean fluorescence intensity of staining is shown. Stained for CD8 APC-Vio770 and the viability stain VIVID.

The above structural data and the CPL data in Figure 1B suggest that the 4C6 T cell will respond to many peptides of 9 amino acids in length with the motif X-H/K/L/M/N/W/Y-X-P/R-L-X-X-X-A/F/I/L/M/V/W. Our proteomic database compiling bacterial species known to be human pathogens (22) contains 678,578 different peptides that incorporate this motif, which further highlights the high capacity for activation of the 4C6 T cell by molecular mimicry. We next examined this aspect in more detail as recently undertaken for HLA A*02:01–restricted insulin-specific TCR InsB4 (22). Testing of the top 20 peptides from a database of viral proteins showed that although 3 of 20 acted as agonists, none of these were more potent than the index PPI_3–11_ sequence (data not shown). Sixteen of the top 20 peptides predicted from the fungal pathogen proteomic database acted as 4C6 agonists, with one from *Cryptococcus* being almost 10,000 times more potent than the index PPI_3–11_ sequence in peptide titrations (Supplemental Figure 6). Searching of the much larger proteomic database from pathogenic bacteria produced the most 4C6 agonists, with 9 of the 20 top predicted peptides being more potent activators of the 4C6 T cell than the index PPI–derived sequence (Figure 4, A and B). All but one of these 9 superagonist ligands included the expected Arg4/Leu5 peg. The remaining peptide, sequence NLLPLAPLF from *Pseudomonas aeruginosa*, included a proline residue in position 4 that showed as a second option at this position in the CPL screen (Figure 1B). It was striking that all the most potent bacterial agonists (RYPRLFGIV, SLPRLFPLL, RYPRLFGIL, and RYPRLFPLL), like the NMPRLFPIV and QLPRLFPLL superagonists (Figure 2B) and the strongest fungal agonist (*Cryptococcus*-derived LLPRLFGLF) (Supplemental Figure 6), contained a central PRLF motif at positions 3–6, suggesting that the ringed amino acid side-chains at positions 3 and 6 might play a role in optimal TCR engagement by supporting the Arg4/Leu5 peg as shown in Supplemental Figure 4A. Indeed, there were 865 peptides within the proteomes of the 1,034 different bacterial species most commonly infecting humans that contained the X-H/K/L/M/N/W/Y-P-R-L-F-X-X-A/F/I/L/M/V/W motif incorporating the position 3–6 PRLF seen in the very strongest agonists and allowing binding to HLA A*24:02. The strongest activating bacterial ligand tested, SLPRLFPLL derived from *Klebsiella oxytoca*, also contained a central PRLF core and was almost 30,000 times more potent than PPI_3–11_ (EC_50_ 5.6 × 10^–11^ M compared with 1.9 × 10^–7^ M with >95% pure peptide; Supplemental Figure 7). The sequence of this peptide, SLPRLFPLL, differs from the LWMRLLPLL PPI_3–11_ sequence at 4 positions, where in each case it uses an amino acid predicted to be preferable in the CPL screen in Figure 1B; this include Leu2 as was the case for QLPRLFPLL superagonist peptide, where it might have effects on the HLA A*24:02–peptide complex similar to those described in Supplemental Figure 4. Tetramer staining of 4C6 T cells with HLA A*24:02 loaded with SLPRLFPLL and another *K*. *oxytoca–*derived peptide (RYPRLFGIV) further confirmed that the 4C6 TCR engaged these pathogen-derived epitopes (Figure 4C). Given the 678,578 bacterial peptides that contain the motif described above and the 18 peptides in Figure 4B that elicited a response, we estimate that in excess of 100,000 peptides found in the proteomes of bacteria known to be pathogenic to humans have the capacity to be recognized by 4C6 T cells with a higher potency than the insulin-derived LWMRLLPLL sequence. Overall, these data highlight the fact that pathogen-derived sequences can act as strong superagonists of T cells bearing the 4C6 TCR through molecular mimicry. It was impossible to study the huge number of peptide sequences within the proteome of pathogenic bacteria with potential to act as superagonists for T cells with 4C6-like TCRs. We therefore chose to focus on the *K*. *oxytoca*–derived peptides as an example for subsequent experiments while remaining mindful of the fact that the chances of any chosen sequence from such a large list of possibilities being biologically relevant to T1D were small. It was first important to establish that candidate sequences could be genuinely processed from bacterial protein and presented at the cell surface in the context of HLA A*24:02.

### K. oxytoca–derived peptides are real T cell epitopes.

**Figure 5:**
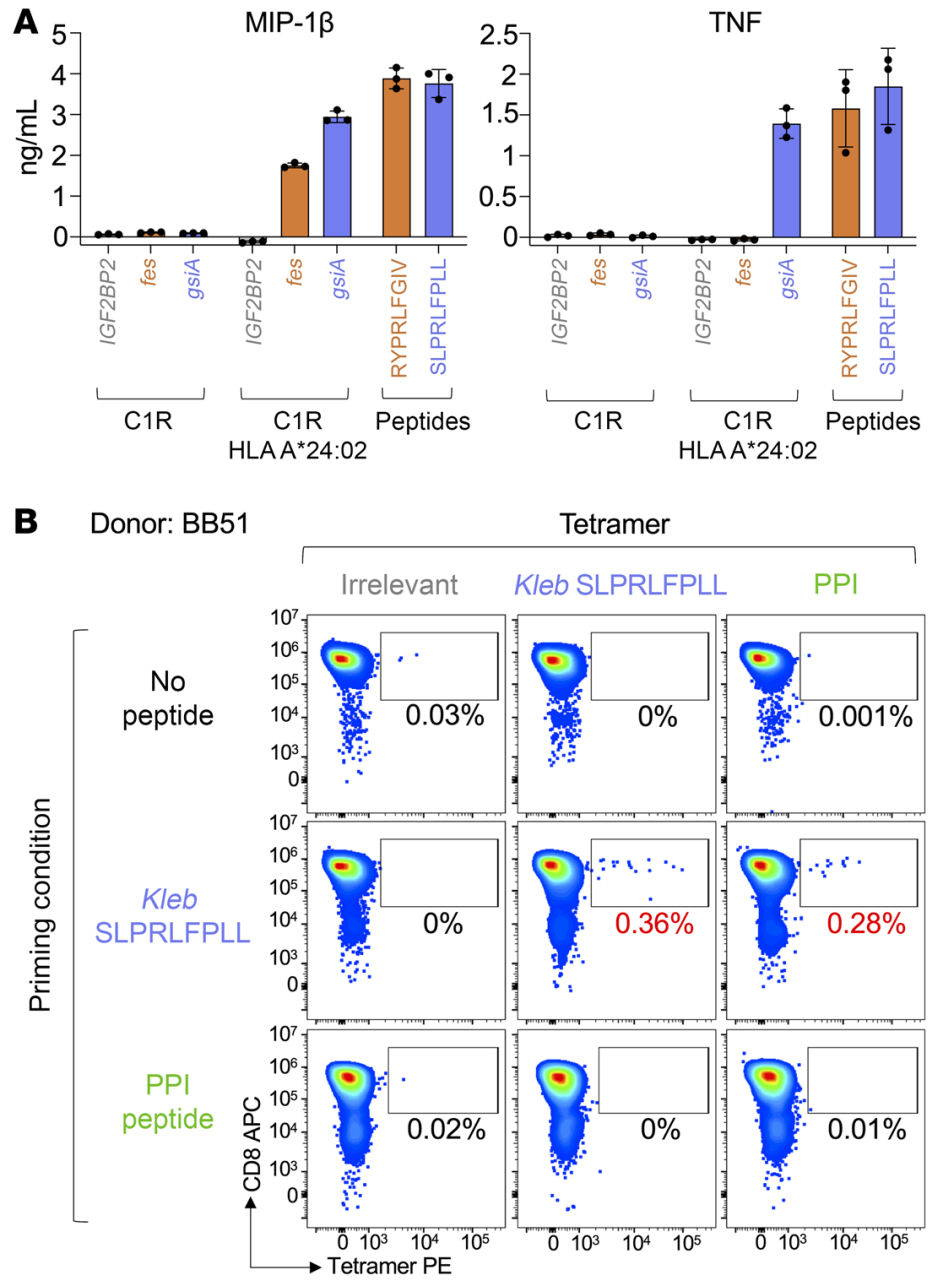
*K*. *oxytoca* peptides are real epitopes and prime PPI-reactive T cells from a healthy donor. (**A**) Genes encoding enterochelin esterase (also known as DUF3327 containing protein) (gene: *fes*) or glutathione ABC transporter ATP-binding protein (gene: *gsiA*) from *K*. *oxytoca* were expressed in C1R cells (with or without HLA A*24:02) using lentivirus, then used in overnight activation assays with PPI LWMRLLPLL peptide–reactive CD8^+^ T cell clone 4C6. Irrelevant protein IMP2 (gene: *IGF2BP2*) was used as a negative control. Peptides (10^–5^ M) RYPRLFGIV from enterochelin esterase and SLPRLFPLL from glutathione ABC transporter ATP-binding protein were used as positive controls. Supernatants used for MIP-1β and TNF ELISA. Error bars represent standard deviation of triplicate conditions. (**B**) Purified CD8^+^ T cells from an HLA A*24^+^ healthy donor (BB51) were primed with *K*. *oxytoca* (*Kleb*) SLPRLFPLL or PPI LWMRLLPLL peptides at 10^–5^ M. Unprimed T cells were cultured without peptide. The lines were stained with HLA A*24:02–irrelevant (AYAAAAAAL), *Kleb* SLPRLFPLL, and PPI tetramers. Percentages are for viable CD3^+^ tetramer^+^ cells.

In order to confirm that *K*. *oxytoca* SLPRLFPLL and RYPRLFGIV peptides could be processed from their respective proteins (amino acid sequences in Supplemental Figure 8A) and presented on HLA A*24:02 to T cells, we lentivirally cotransduced HLA A*24:02 and *fes* or *gsiA* genes from *K*. *oxytoca* into 3 different HLA A*24:02-negative cell lines (C1R, THP-1, and A549), thereby creating “surrogate” infected cells. Transduction with human *IGF2BP2* was used as a control. Each *K*. *oxytoca* gene transductant was capable of activating the 4C6 CD8^+^ T cell clone in the presence of HLA A*24:02, with similar results for all 3 cell lines used (Figure 5A and Supplemental Figure 8B). The *gsiA* gene, encoding glutathione ABC transporter ATP-binding protein, which contains the SLPRLFPLL epitope, was capable of inducing MIP-1β and TNF to levels comparable with 10 μM exogenous *Klebsiella* peptide (Figure 5A and Supplemental Figure 8B). The *fes* gene, encoding enterochelin esterase protein (RYPRLFGIV peptide), induced MIP-1β but not TNF; and as MIP-1β is a more sensitive T cell assay output than TNF (35, 36), these data could be a reflection of the reduced potency of 4C6 toward the RYPRLFGIV peptide compared with SLPRLFPLL (Figure 4), or differences in the processing and presentation of the epitopes from their respective proteins. As SLPRLFPLL peptide from *K*. *oxytoca* was the most potent of the pathogen derived peptides at activating 4C6 (Figure 5A and Supplemental Figure 8B) and exhibited enhanced stimulation of 4C6 compared with the *K*. *oxytoca* RYPRLFGIV peptide in the epitope validation experiments (Figure 5A), we concentrated on SLPRLFPLL for downstream experiments.

### Klebsiella and PPI cross-reactivity exists in other T1D patients.

We next looked at 2 other HLA A*24^+^ donors with T1D (CCPO-1406 and T1D-12) to establish whether the PPI cross-reactivity with *Klebsiella* observed in the patient that 4C6 was grown from (14) existed in other patients. Magnetic enrichment with PPI-LWMRLLPLL tetramers from the PBMCs of patient CCPO-1406, or cultured T-cells from patient T1D-12, revealed T-cells that also stained with *Klebsiella* SLPRLFPLL tetramers, suggesting that the cross-reactivity we describe is not exclusive to the patient we derived the 4C6 T-cell from (Supplemental Figure 8C).

**Figure 6:**
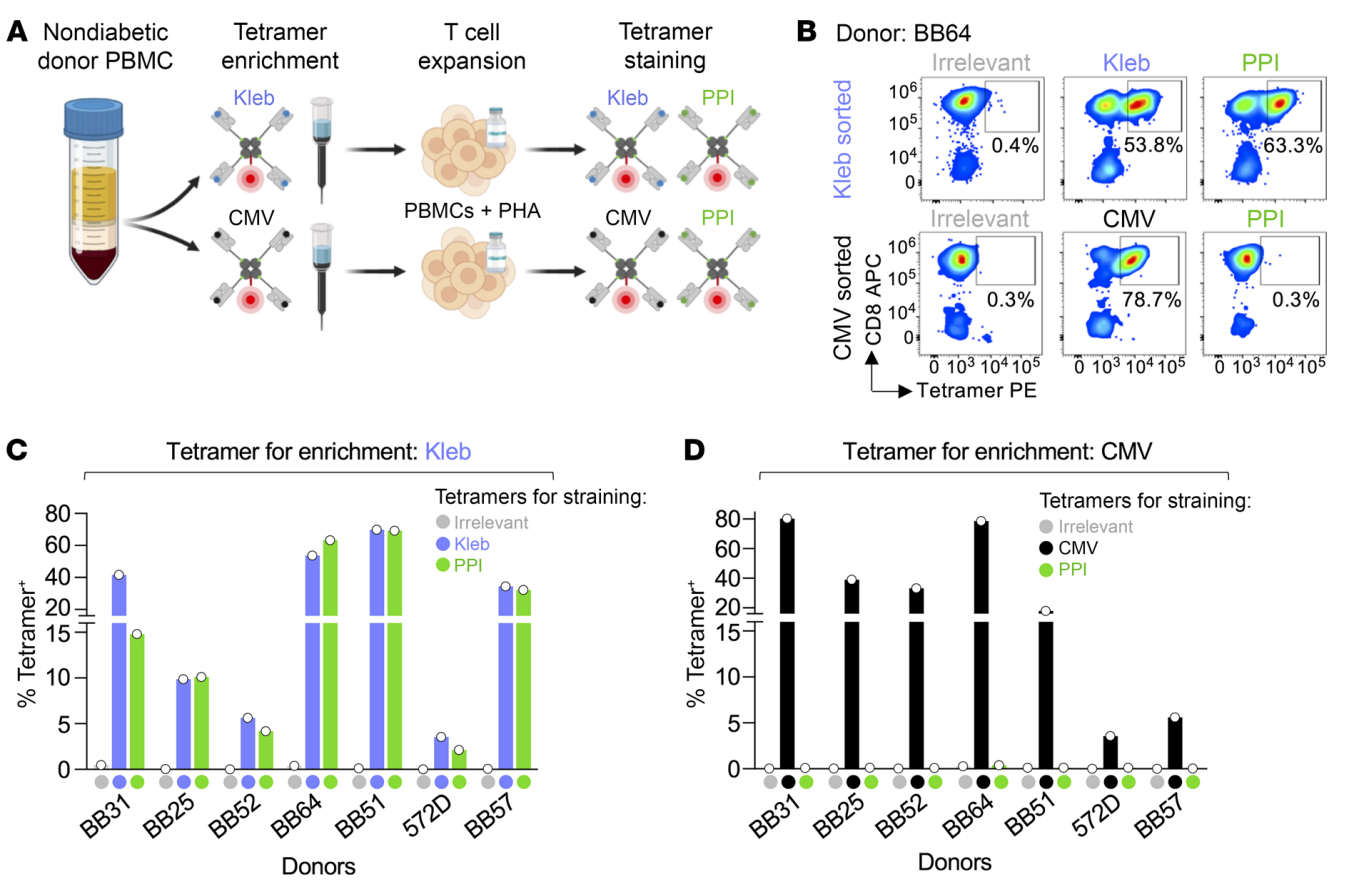
*Klebsiella* tetramers co-select PPI-specific T cells in nondiabetic donors. (**A**) PBMCs from 8 HLA A*24^+^ healthy donors were enriched in parallel with HLA A*24:02 *Klebsiella* SLPRLFPLL or CMV-AYAQKIFKIL PE-conjugated tetramers and anti-PE magnetic beads. After 2 weeks of expansion with allogeneic PBMCs and PHA, the T cell lines were stained with irrelevant (HLA A24:02 AYAAAAAAL; not shown in the schematic), PPI (LWMRLLPLL), and *Klebsiella* or CMV (depending on the enrichment) tetramers. (**B**) Tetramer staining of enriched T cell lines from donor BB64. Percentage tetramer^+^ is shown. (**C**) Percentage of *Kleb*, PPI, and irrelevant tetramer staining for *Kleb* tetramer–enriched lines from 7 of 8 donors. Donor BB72 did not have *Kleb* T cells, so those data are not shown. Donor BB64 staining is shown in **B**. Performed as single-staining conditions. (**D**) Percentage of CMV, PPI, and irrelevant tetramer staining for CMV tetramer–enriched lines from 7 of 8 donors. Donor BB64 staining shown in **B**. Performed as single-staining conditions.

Next, we used the *K*. *oxytoca* SLPRLFPLL epitope as an example of a naturally occurring superagonist peptide sequence and were interested in whether T cell cross-reactivity between *Klebsiella* and PPI also exists in healthy HLA A*24^+^ donors. We primed CD8^+^ T cells from HLA A*24^+^ donor BB51 with the *Klebsiella*-derived sequence and showed that the resultant T cell line also bound tetramers made with the PPI epitope (Figure 5B). We then used tetramers and magnetic sorting (25) to make enriched T cell lines from donor BB51 and other HLA A*24^+^ nondiabetic healthy donors. *Klebsiella* SLPRLFPLL- and CMV AYAQKIFKIL–binding T cells were isolated from donor PBMCs with tetramer and then cultured so that there were enough cells to work with (Figure 6A). The resulting T cell lines stained with respective enriching (*Klebsiella* or CMV) and PPI tetramers. *Klebsiella* tetramer–enriched cell lines from 7 of 8 donors stained positive for *Klebsiella* and PPI tetramers (Figure 6, B and C, and Supplemental Figure 9), whereas none of the CMV tetramer–enriched lines from the same donors stained with PPI tetramer (Figure 6, B and D, and Supplemental Figure 9). Cells derived from the eighth donor (BB72) did not stain with *Klebsiella* or CMV tetramers (data not shown). It is possible that the donor was not HLA A*24^+^ based on the cross-reactivity of the antibody used for HLA typing (antibody clone 4i94 also binds HLA A*23). The *Klebsiella* tetramer also coselected T cells that stained with the second *Klebsiella* epitope, RYPRLFGIV (Supplemental Figure 9). Overall, in cells from 5 of 7 donors (BB25, BB52, BB64, BB51, and BB57), the percent tetramer^+^ staining with the *Klebsiella* (SLPRLFPLL and RYPRLFGIV) and PPI (LWMRLLPLL) epitopes was similar; whereas for donor BB31, the enriching SLPRLFPLL epitope stained more (41.6%) than with RYPRLFGIV (15.3%) and LWMRLLPLL (14.8%) tetramers. Similarly, cells derived from donor 572D stained more with SLPRLFPLL (3.6%) than with RYPRLFGIV (1.12%) and LWMRLLPLL (2.13%) (Figure 6, B and C, and Supplemental Figure 9). This suggests that in the case of all donor cell lines from which we were able to culture *Klebsiella* peptide–specific T cells, these T cells could also engage the PPI-derived peptide LWMRLLPLL in the context of HLA A*24:02. Indeed, across the cohort, most of the *Klebsiella* peptide–reactive T cells also recognized the PPI epitope.

### Healthy donor Klebsiella epitope–enlisted T cells kill surrogate pancreatic β cells.

**Figure 7:**
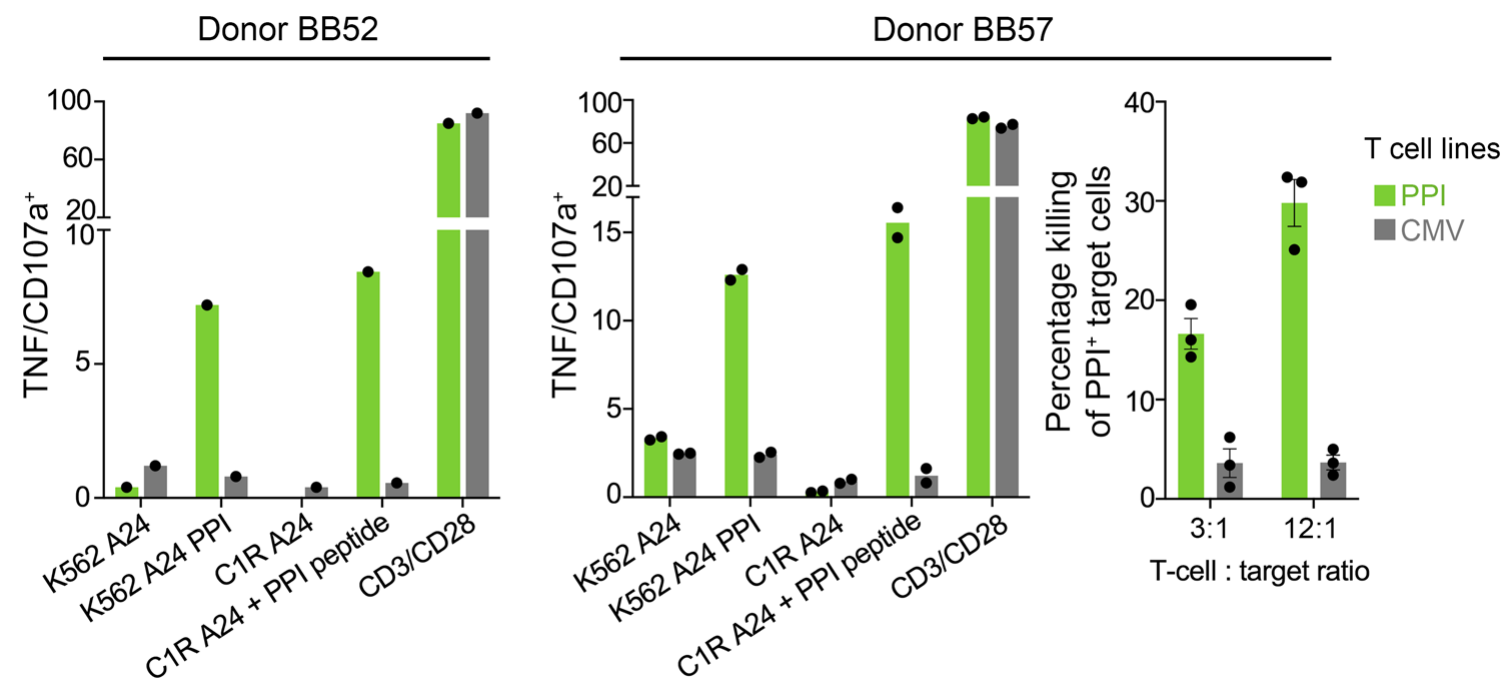
T cell lines enriched with *Klebsiella* peptide recognize and kill HLA A*24:02^+^ target cells expressing PPI. T cell lines created from healthy HLA A*24^+^ donors by enrichment with HLA A*24:02 *Klebsiella*–SLPRLFPLL tetramers were found to be cross-reactive with LWMRLLPLL from PPI. CMV (AYAQKIFKIL) T cell lines from the same donors were used in parallel. Left and middle: T cells lines were incubated with K562s expressing HLA A*24:02 (K562 A24) with or without PPI (K562 A24 PPI). Reactivity toward 10^–6^ M PPI peptide was assessed using C1R HLA A*24:02 (C1R A24) as antigen-presenting cells. CD3/CD28 beads used as a positive control for T cell activation. Data points shown for duplicate conditions. Right: T cell lines from BB57 incubated (6 hours) with chromium 51–labeled K562 A24 and K562 A24 PPI cells. Background killing with K562 A24 cells was subtracted from killing with K562 A24 PPI cells. Performed in triplicate with error bars showing SEM.

The *Klebsiella* peptide T cell lines from donors BB52 and BB57 were tested in activation assays with surrogate pancreatic islets cells (K-562 cells expressing HLA A*24:02 and PPI), which were recognized by the T cells at levels similar to those seen with exogenous PPI peptide (Figure 7). We also tested the *Klebsiella* and CMV lines from donor BB57 in cytotoxicity assays, which showed that the PPI-reactive cells induced with a *Klebsiella* peptide were able to kill surrogate pancreatic islet cells (Figure 7). Overall, these data demonstrate that T cells that can be primed and grown from healthy donor PBMCs using a peptide sequence from *K*. *oxytoca* also recognized and killed surrogate pancreatic cells through the PPI epitope. The common cross-recognition of insulin by *Klebsiella* peptide–derived T cells suggested that there may be a common mode of binding of these peptides across the population and the existence of a “public” TCR chain, so we next examined the sequence of these cross-reactive TCRs in multiple donors.

### Klebsiella and PPI cross-reactive TCRs from healthy donors exhibit similarities to 4C6.

TCR sequencing of *Klebsiella* and PPI tetramer T cell line populations from 4 of the healthy donors (BB51, BB52, BB57 and BB64) revealed seven cross-reactive TCRs, with 1–4 TCRs per donor (Supplemental Figure 10). There was little evidence of monospecific TCRs for only the *Klebsiella*-derived peptide used to initially select the T cells, as 96%–100% of the *Klebsiella* TCRs from each donor also bound the PPI tetramer (Supplemental Figure 10). This is consistent with the similar percentages of tetramer staining for the *Klebsiella* and PPI peptides seen for these donors, as shown in Figure 6C. There were no *TRAV/TRAJ* or *TRBV/TRBJ* gene bias across the TCRs from the healthy donors but there were some potential similarities with key peptide-HLA contact residues of the 4C6 TCR as indicated (Supplemental Figure 10). We compared key contact residues of the 4C6 TCR from CDR1 (Asp27α, Ser29α, Ser30α, Tyr32α, and Arg32β) and CDR3 (Pro93α, Ser94α, Gly95α, Asn96α, Thr97α, Gly98α, His98β, and His99β) with the healthy donor TCRs. The 4C6 TCR and one of the TCRs from the healthy donors (BB57) both utilize *TRAV5*, so the CDR1α amino acid residues of the 4C6 TCR (Asp27α, Ser29α, Ser30α and Tyr32α) that bind the PPI peptide may also assist in epitope recognition by the BB57 TCR (Supplemental Figure 10). Similarities in CDR3α between the 4C6 and healthy donor TCRs included a non-germline proline residue in donors BB52 and BB64 (Supplemental Figure 10). This Pro93α residue of the 4C6 TCR forms 3 interactions with Arg4 of the PPI peptide (Figure 3E). There were other similarities associated with CDR3α, suggesting that the mode of TCR binding to HLA A*24:02–LWMRLLPLL may be similar across different donors (as indicated in Supplemental Figure 10), but further sequence alignments or structures would be required in order to designate any similarities as a TCR “motif.”

## Discussion

CD8^+^ T cells use their clonotypic TCR to interrogate the proteome of other cells to find and eliminate anomalies that arise because of cellular transformation or infection. This ingenious system is made possible by the HLA-I presentation pathway, which presents short peptides from intracellular proteins at the cell surface. Every human has approximately 4 × 10^11^ T cells, each expressing one of approximately 10^8^ unique TCRs, a number that is dwarfed by the more than 10^17^ foreign peptides that could theoretically be encountered (37–39). Pathogens evolve much faster than their mammalian hosts and could quickly adapt to exploit T cell blind spots; so to be effective, T cell immunity must cover all possibilities (19). Simple arithmetic dictates that a comprehensive immune system requires T cells to be highly cross-reactive (19, 39). Indeed, we previously showed that the T1D patient–derived, HLA A*02:01–restricted, PPI-specific CD8^+^ T cell clone 1E6 could recognize well over 1 million different 10-mer peptides with a potency equivalent to, or greater than, that of the index PPI sequence used to destroy human β cells (20).

Here we investigated the T1D patient–derived TCR 4C6, which kills human β cells via presentation of PPI_3–11_: sequence LWMRLLPLL by HLA A*24:02, an HLA-I known to be enriched in patients with T1D (7). SPR binding data showed that the 4C6–HLA A*24:02–LWMRLLPLL interaction was relatively weak (*K_D_* ~130 μM) but nonetheless higher than usually observed for autoimmune TCRs (25). Our initial, extensive attempts to crystallize the 4C6 TCR with its cognate PPI-derived ligand failed. Functional analysis of the 4C6 T cell clone showed that it exhibited a preference for peptides of 9 amino acids in length. The data from a 9-mer CPL screen was used to generate a superagonist peptide, QLPRLFPLL, that was more than 100,000 times more potent at activating 4C6 T cells than the natural insulin-derived epitope in titration assays when using peptides of >95% purity. The QLPRLFPLL superagonist peptide bound to the 4C6 TCR with more than 20-fold-higher affinity than the natural PPI-derived peptide epitope. The higher affinity of interaction with the superagonist peptide allowed us to generate 4C6 TCR–ligand crystals (29). As the peptide in TCR:peptide-HLA complexes is sandwiched between the TCR and HLA and not solvent exposed, we reasoned that this might allow us to use the QLPRLFPLL-containing crystals as seeds for growing crystals of the much weaker 4C6–HLA A*24:02–LWMRLLPLL interaction. Seeds expedite the formation of a crystal lattice from a supersaturated solution by providing a preformed lattice to precipitate upon, removing reliance on random diffusion for nucleation. Seeding allowed us to generate 4C6–HLA A*24:02–LWMRLLPLL cocrystals that diffracted at 2.48 Å. This technique — in which TCRs in complex with strong superagonist ligands, binding at a *K_D_* range of 0.1–10 μM (25, 40) are used to seed crystals with weaker ligands — might provide a way to generate structures of TCRs with other autoimmune antigens that tend to bind weakly (*K_D_* >100 μM) (25), making them notoriously difficult to crystallize.

The 4C6–HLA A*24:02–LWMRLLPLL structure showed that residues Arg4 and Leu5 of PPI_3–11_ protruded toward the TCR to form a peg-in-hole network of bonding interactions that accounted for 72% of contacts between the TCR and peptide, including all the hydrogen bonds. The greater than 20-fold-higher affinity with the QLPRLFPLL superagonist peptide was likely due to a combination of factors, including greater stabilization of the Arg4/Leu5 peg because of the optimal Leu2 peptide anchor; increased intrapeptide bonds between Pro3 and Phe6 compared with Met3 and Leu6 in the index peptide; as well as enthalpically favorable thermodynamics, promoting a net increase in electrostatic bond formation during binding. It is also possible that the structurally silent peptide anchor differences allosterically modulated TCR binding, as described for position 2 variants of an HLA A*02:01–restricted epitope from gp100 (41). Overall, the Arg4/Leu5 peg served as a TCR binding “hot spot” in a manner analogous to the GPD motif in 10-mer peptides recognized by the HLA A*02:01–restricted 1E6 TCR (31, 42) and suggested that T cells expressing the 4C6 TCR might be capable of responding to a large number of 9-mer peptides in the context of HLA A*24:02.

Biased sampling of the 9-mer peptide universe estimated that more than 1,000,000 peptides might act as agonists of the 4C6 T cell clone with a potency equivalent to or better than that of the PPI_3–11_ index sequence. This estimate is a similar to that we produced for the recognition of 10-mer peptides in the context of HLA A*02:01 by the 1E6 TCR (20) and in line with theoretical predictions made more than 25 years ago by Mason (39). Our biased sampling of our proteome database of bacteria that are known human pathogens suggested that it contains more than 100,000 peptide sequences that would act as more potent agonists for the 4C6 T cell than the insulin index sequence. Searching of pathogen proteomics databases with the algorithm developed by Szomolay and colleagues (23) and recently accelerated and refined to run on graphics processing units (22) revealed several pathogen-derived amino acid sequences that acted as very strong agonists of the 4C6 T cell. One peptide, derived from the proteome of *K*. *oxytoca*, was almost 30,000 times more potent than the index PPI-derived sequence. The two strongest agonists of the 4C6 TCR tested contained an optimal leucine at position 2 and a PRLF motif at positions 3–6. Bayesian refinement of epitope searching algorithms to include these findings could result in discovery of further strong pathogen-derived agonists for 4C6 TCR-expressing T cells.

We next examined whether cross-reactivity between bacterial and PPI peptides might be a common feature in HLA A*24^+^ healthy donors using the *K*. *oxytoca*–derived sequence SLPRLFPLL as an example. We were able to prime PPI-reactive T cells from the PBMCs of an HLA A*24^+^ healthy donor using the *Klebsiella*-derived sequence SLPRLFPLL. Additionally, HLA A*24:02–SLPRLFPLL tetramers were used to isolate T cells from 7 of 8 donors whose PBMCs stained with antibody clone 4i94 (specific for HLA A*24 and A*23). In all cases the SLPRLFPLL-specific T cell lines also stained with tetramers made with the PPI_3–11_ sequence, which suggested that such cross-reactivity can occur commonly. We had enough cells from two donors to show that the SLPRLFPLL-generated T cell lines, but not those generated with the CMV-derived epitope AYAQKIFKIL, reacted toward and killed HLA A*24:02^+^ targets expressing PPI.

In summary, we demonstrate the molecular mechanism by which a T1D patient–derived TCR engages the PPI_3–11_ epitope to kill human β cells and show how focused binding with residues 4 and 5 of the LWMRLLPLL epitope allowed this TCR to strongly interact with some pathogen-derived peptide sequences. These results provide a mechanism by which T cells with such TCRs might become “armed and dangerous” and trigger autoimmune attack via molecular mimicry (Supplemental Figure 11).

## Methods

All procedures are described in detail in Supplemental Methods.

### Sex as a biological variable.

Sex was not considered as a biological variable, and we report similar results for 7 of 8 nondiabetic donors and all 3 participants with T1D.

### Study approval.

Donors recruited via the Welsh Blood Service gave written informed consent as part of the donation procedure, and samples were used under local ethical approval granted by the Cardiff University School of Medicine Research Ethics Committee (reference 18/56). Participants with T1D were recruited to the clinical study Characterisation of the Immune Response to SARS-CoV-2 Infection and Other Common Human Pathogens in Type 1 Diabetes following receipt of written informed consent (IRAS ID 253888; ClinicalTrials.gov NCT04729452). Ethical permissions were granted by Wales REC5 (Bangor University, Bangor, United Kingdom), and the study sponsor was the Cwm Taf Morgannwg University Health Board, Mountain Ash, United Kingdom.

### Data availability.

Structural data are available via the Protein Data Bank (https://www.rcsb.org; PDB entries 7NMD, 7NME, 7MNF and 7MNG as listed in Supplemental Table 1. Biological data sets are presented in the Supporting data values file. Other data are available from the corresponding author upon reasonable request.

## Author contributions

AKS and GD conceived the study. GD, AB, AW, HT, JRH, CR, SAEG, AF, LRT, TM, NO, KT, MSH, B Szomolay, OBS, and DKC designed research studies, conducted experiments and acquired data. GD, AB, AW, TW, B. Szomolay, HT, HAVDB, MP, DKC, PJR, and AKS analyzed the data. DKV and MP provided critical reagents. AKS, GD, AW, B Szomolay, HAVDB, and PJR wrote the manuscript. LCJ was the chief clinical investigator; designed and the clinical protocol, paperwork and ethical application for the work involving patients with type I diabetes; recruited patients, and carried out the clinical protocol. Clinicians SJ, NDS, and THH were involved in recruiting patients from clinics. More than 80% of the data herein were generated by GD, AB, and AW, who were assigned equal contribution. GD performed or supervised all the cellular experiments shown. AB undertook the SPR data and generated crystals and x-ray data of the 4C6 TCR with PPI and superagonist peptide. AW solved the 4C6 TCR complex structures, set crystals, solved the structures for the superagonist monomer, and made the structural figures.

## Supplementary Material

Supplemental data

Supporting data values

## Version 1 09/17/2024

Electronic publication
