# Supplementary material for: HLA A*24:02–restricted T cell receptors cross-recognize bacterial and preproinsulin peptides in type 1 diabetes
Source: J Clin Invest. 2024 Sep 17;134(18):e164535. doi: 10.1172/JCI164535 (PMC11405051; doi:10.1172/JCI164535)
Supplement: Supplemental data [file jci-134-164535-s114.pdf]

Supplementary Materials for

HLA A*24:02-restricted T-cell receptors cross recognise bacterial and preproinsulin peptides in type 1 diabetes

Garry Dolton[1], Anna Bulek[1], Aaron Wall[1], Hannah Thomas[1], Jade R. Hopkins[1], Cristina Rius[1], Sarah A.E. Galloway[1], Thomas Whalley[1], Li Rong Tan[1], Théo Morin[1], Nader Omidvar[1], Anna Fuller[1], Katie Topley[1], Md Samiul Hasan[1], Shika Jain[2], Nirupa D'Souza[2], Tom Hodges-Hoyland[3], the TIRID Consortium, Owen B. Spiller[1], Deborah Kronenberg-Versteeg[4], Barbara Szomolay[5], Hugo A. van den Berg[6], Lucy C. Jones[1,2], Mark Peakman[4], David K. Cole[1], Pierre J. Rizkallah[1] and Andrew K. Sewell[1,5]

[1]Division of Infection and Immunity, Cardiff University School of Medicine, Cardiff, Wales, UK

[2]Cwm Taf Morgannwg University Health Board, Princess of Wales Hospital, Wales, UK

[3]Ashgrove Surgery, Pontypridd, Wales, UK

[4]Department of Immunobiology, King's College London, UK

[5]Systems Immunology Research Institute, Cardiff University Cardiff, Wales, UK

[6]Warwick Systems Biology Centre, University of Warwick, Coventry, UK

Correspondence to Andrew Sewell: sewellak@cardiff.ac.uk

This PDF file includes:

- Supplementary Materials and Methods
- Supplementary Figures S1 to S11
- Supplementary references 43-54
- **Supplemental Acknowledgements**: TIRID (T-cell Immune Response In Diabetes) Consortium.
- **Supplementary Table 1:** X-ray Crystallography statistics for presented 3D structures
- **Supplementary Table 2:** Molecular contacts in the 4C6-HLA A*24:02-LWMRLLPLL cocrystal structure
- **Supplementary Table 3:** Molecular contacts in the 4C6-HLA A*24:02- QLPRLFPLL cocrystal structure

Supplementary Materials and Methods

Non-diabetic Donors

Blood samples from healthy donors were sourced from the Welsh Blood Service (Velindre NHS Trust, Wales, UK) as EDTA treated ‘buffy coats’ and ethical approval granted by the School of Medicine Research Ethics Committee (reference 18/56). Each buffy coat was seronegative for HIV-1, HBV and HCV. Blood and cells derived thereof were handled in accordance with Cardiff University guidelines in alignment with the United Kingdom Human Tissue Act 2004. Buffy coat from the WBS was diluted 1:1 with R10 (RPMI-1640 supplemented with 10% heat-inactivated FBS, 100 U/ml penicillin, 100 µg/mL streptomycin and 2 mM L-glutamine (all Merck)) and placed on a Cole-Parmer^{TM} Stuart^{TM} roller-mixer overnight at 9-11 rpm and room temperature. The following morning the blood was further diluted 2:1 (blood:RPMI-1640) then PBMCs separated using conventional density gradient centrifugation with Sigma-Aldrich Histopaque 1077 (Merck). Red blood cells were removed using lysis buffer (155 mM NH_4Cl, 10 mM $KHCO_3$ and 0.1 mM EDTA, pH7.2-7.4) for 10 min at 37°C. PBMCs were cryopreserved in 90% FBS and 10% DMSO using a controlled rate freezing device (either CoolCell, Biocision or “Mr Frosty”, Nalgene) at -80°C. Cells were moved within 48 h to vapour phase liquid nitrogen for long term storage. Cells were thawed rapidly in a 37°C water bath before being resuspended in pre-warmed R10 medium and centrifuged at 300 x *g* for 5 min. Defrosted PBMCs were treated at this point with 50 µg /mL DNase-I (Roche, Burgess Hill, U.K.) at 37°C in R10 for 20 min before being used for assays.

Participant samples

Venous blood donated by individuals with type 1 diabetes (CCPO-1406 and T1D-12) participated in the study ‘characterisation of the immune response to SARS-CoV-2 infection and other common human pathogens in type 1 diabetes’ IRAS: ID 253888, which is registered on clinicaltrials.gov registry. Ethical permissions were granted by Wales REC5 and the study Sponsor was Cwm Taf Morgannwg University health board. The study was performed in accordance with the ethical standards as laid down by the 1964 Declaration of Helsinki and its later amendments and is a prospective, observational study. Eligible participants were convalescent patients with a diagnosis of type 1 diabetes made in the last 3 years at Cwm Taf Morgannwg University health board, Cardiff and the Vale University health board (Noah’s Ark Children’s Hospital) and Imperial College Healthcare NHS Trust. Participants were recruited between December 2020 and December 2023 and aged 8 to 43 years of age, without any immunological conditions and had not received immune modulating medications or transfusions of whole blood or blood derivatives in the previous 3 months prior to venous blood sample donation. Following written informed consent, a sample of venous blood was donated (30 mL in adult participants and 20 mL in children, according to the participants weight and the formula 0.8 mL/kg) in Becton Dickinson vacutainers containing EDTA. Samples were anonymised prior to transfer to the Cardiff University laboratory on the same day of sampling with MTA, placed on a roller overnight, then separated using conventional density gradient centrifugation as above, but without pre-dilution nor red blood cell lysis. PBMCs were used immediately for assays without cryopreservation.

HLA A*24 typing of donors with antibody

For the HLA typing of healthy donors an unconjugated anti-HLA A*24 antibody (catalog number H6098-29, clone 4i94, United States Biological, Salem, MA, USA) was used with a goat anti-mouse PE conjugated secondary antibody (catalog number 550589, BD Biosciences). The primary antibody also binds to HLA A*23, so the 1/8 donors that we failed to generate HLA A*24 restricted T-cells from may have been HLA A*23$^+$ and not HLA A*24$^+$.

4C6 T-cell clone

The HLA A*24:02-restricted CD8$^+$ T-cell clone 4C6 recognises a naturally-processed peptide fragment of preproinsulin (PPI_{3-11}, LWMRLLPLL). The clone was derived from a recently-diagnosed HLA A*24:02$^+$ T1D patient (14). The 4C6 T-cell clone was cultured in T-cell media (RPMI-1640 supplemented with 10% heat-inactivated FBS, 100 U/ml penicillin, 100 μg/mL streptomycin, 2 mM L-glutamine, 25 ng/ml IL-15 (Miltenyi Biotech, Bergisch Gladbach, Germany), 200 IU/mL IL-2 (Proleukin®; Prometheus, San Diego, CA), 1X non-essential amino acids solution, 1 mM sodium pyruvate and 10 mM HEPES buffer (all from Merck, St Louis, Missouri, USA, unless stated otherwise). Every 2-3 weeks, 4C6 (1×10^6) was expanded using 1.5×10^7 irradiated (3100 Gy) PBMCs from three allogenic donors provided by the Welsh Blood Service and 1 μg/mL of L-phytohemagglutinin (Pan Biotech, Wimborne, UK) as previously described (43, 44) in T-cell media with 20 IU/mL of IL-2. The 4C6 TCR sequence is shown in **Supplementary Figure 1**. Mycoplasma testing was conducted using a MycoAlert® mycoplasma detection kit according to the manufacturer's instructions (Lonza, Basel, Switzerland).

Antigen presenting cells

The following cell lines were sourced locally with ATCC references included to act as a resource for culture and validation guidelines: the B-lymphoblastoid C1R (ATCC® CRL-1993™), acute monocytic leukaemia THP-1 (ATCC® TIB-202™) and lung carcinoma A549 (ATCC® CCL-185™), with each being cultured in R10 media. C1R and THP-1 cells were grown in suspension and split 1:10 once or twice a week. A549 are adherent and passaged by detachment with D-PBS and 2 mM EDTA and split 1:10-1:20 once a week. C1R, THP-1 and A549 cells are naturally HLA A*24:02 negative and therefore transduced with *HLA A*24:02* to act as antigen presenting cells for the study. The *fes* and *gsiA* genes from *Klebsiella oxytoca* strains, encoding Enterochelin esterase containing the RYPRLFGIV sequence and *gsiA* encoding Glutathione ABC transporter ATP-binding protein containing the SLPRLFPLL sequence respectively (**Supplementary Figure 8A**) were lentivirally transduced for epitope validation experiments (see below). Briefly, codon optimised *HLA A*24:02, fes* and *gsiA* genes were synthesised (Geneart, Life Technologies, Carlsbad, California, US) and cloned in-house to a third-generation lentiviral plasmid (p), SnapFast (pSF) (Oxgene, Oxford Genetics Ltd, Littlemore, Oxford, UK). The *fes* and *gsiA* genes were expressed with a rat (r) CD2 co-marker: XbaI-Kozak-*gene*-XhoI-P2A-rCD2-SalI-Stop, whereas HLA A*24:02 did not require the rCD2 co-marker as it is expressed at the cell surface: XbaI-Kozak-*gene*-Stop-SalI-Stop. C1R cells were also transduced with the *IGF2BP2* gene encoding IMP2 as previously described (45) with a rCD2 co-marker, to act as an irrelevant protein for the *Klebsiella* epitope validation experiments. For transfection, pSF (1.52 μg), envelope plasmid (pMD2.G;

0.72 µg) and packaging plasmids (pMDLg/pRRE; 1.83µg and pRSV-REV; 1.83µg) were mixed in 300 µL of Opti-MEM, reduced serum medium (Thermo Fisher Scientific) followed by mixing with 1 µg/mL Polyethylenimine (PEI; Merck) at a 3:1 PEI: plasmid ratio. Plasmid/PEI mixtures were incubated at room temperature for 15 min, added dropwise to locally sourced HEK293T cells (80% confluence in one well of a 6-well plate) and incubated at 37°C in a 5% CO_2 humidified atmosphere. The supernatants containing lentiviral vectors were harvested, passed through a 0.4 µm filter and used immediately for transduction. Cells (0.1-0.2 x10^6 per well of a 24 well plate) were transduced by spinfection with 1 mL of viral supernatant and 1 mL of media at 800 x *g* for 1.5 h at room temperature with 0.5 mg/mL of LentiBOOST (Sirion Biotech, Cambridge, MA, USA), then incubated at 37°C overnight and media replaced the following morning. For C1R cells and transduction of *HLA A*24:02*, expression was confirmed one week post transduction by staining with anti HLA A*24 PE conjugated antibody (catalog number LS-C179732-100, clone 17A10, LsBIO, Seattle, WA, US) and a clone procured by limiting dilution in 96U well plates. Expression of HLA A*24 for THP-1 and A549 cells was confirmed using anti-HLA A*24 antibody clone 4i94 as above, and cells sorted via a BD FACS Aria III (Central Biotechnology Services, Cardiff University) to achieve cell lines with >95% HLA A*24 expression. Cell lines expressing *gsiA*, *fes or IGF2BP2* were enriched via the rCD2 co-marker using 0.02 mg of a mouse anti-rCD2 PE conjugated antibody (catalog number 201305, clone OX-34, BioLegend) per 0.1-10 x10^6 cells in 50-100 µL of MACS buffer (D-PBS, 0.5% BSA and 2 mM EDTA), followed by magnetic enrichment using anti-PE microbeads according to the manufacturer's instruction (Miltenyi Biotec). K-562 cells (ATCC® CCL-243™), expressing HLA A*A24 and preproinsulin were generated before and cultured in R10 as suspension cells with selection antibiotics (14). All cell lines were tested for mycoplasma, as above.

Sizing scan and PS-CPL screening

The peptide length-preference of the 4C6 T cell clone was determined by 'sizing scan' as previously described (21). 4C6 T-cells were rested overnight in R5 media (as for R10 but with 5% FBS) to reduce spontaneous activation, followed by incubation in the presence of C1R-HLA A*24:02, with peptide mixtures of defined length and random amino acid sequence ($X^{8\text{-}14}$ in which X is any of the 20 proteogenic L-amino acids, excluding cysteine) in R5 media overnight at 37°C and 5% CO_2. MIP-1β production was measured by enzyme-linked immunosorbent assay (ELISA, R&D Systems, Bio-Techne, Minneapolis, MN, US) as a proxy for T cell activation as previously described (22). Peptide mixtures were synthesised at >40% purity (Pepscan Presto, Lelystad, Netherlands) and stored at -80°C in DMSO at 40 mM. Mixtures were used in assays at a concentration of 1 mM. Following conformation of the preference of 4C6 T-cells for 9-mer peptides, nonamer PS-CPL screening was performed using libraries synthesised at >40% purity as described previously (22, 23, 43). Libraries were stored at -80°C in DMSO at 20 mM. For assays, 6×10^4 C1R-HLA A*24:02 were pulsed with 100 µM of each CPL library mixture for 2 h at 37°C. Following this, 3×10^4 4C6 T cells were co-incubated with the pulsed C1R-HLA A*24:02 cells overnight at 37°C and 5% CO_2. Assay conditions were performed in duplicate. Supernatants were harvested the following morning and assayed for the presence of MIP-1β, as above, by ELISA.

Positional scanning combinatorial libraries (PS-CPL) for identification of agonist peptides

CPL-based importance sampling, as described in (20), was used to sample peptides from the entire peptide universe with bias toward good agonists. Additionally, PS-CPL screening data from 4C6 T-cells was used to probe a human pathogen proteome database to produce a ranked list of likely peptide ligands, using a publicly accessible webtool pioneered by Szomolay *et al.* (23) and refined by Whalley and colleagues (22). Top-ranking candidate peptides were ordered in crude form (>40% purity; GLS Biochem, Shanghai, China). Confirmed peptide agonists were subsequently ordered in pure form (>95% purity, Peptide Synthetics, Hampshire, UK). Reactivity of 4C6 T-cells to candidate peptides was assessed by ELISA as previously described (43). Assays conditions were performed in duplicate and EC_{50} values were generated using GraphPad Prism software analysis (v.9.0.1).

pMHC tetramer assembly and staining

Soluble, biotinylated peptide-HLA-A*24:02 monomers were produced as described previously (31). Monomers were tetramerised via the step-wise addition of PE-conjugated streptavidin (Life Technologies, Carlsbad, California, US) at a pMHC:streptavidin molar ratio of 4:1. Protease Inhibitor (Merck) and PBS were added to give a final pMHC tetramer concentration of 0.1 mg/mL (with regard to the pMHC component). For staining of the 4C6 T-cell clone, 0.5 µg of tetramer (with respect to the pMHC component) was used to stain 5×10^4 T cells. Irrelevant HLA A*A24:02 AYAQKIFKIL (CMV, $IE1_{1248-1257}$) tetramers were used as a negative control for the 4C6 clone. Some stains required use of an optimised tetramer staining protocol (24, 46), involving pre-incubation with 50 nM protein kinase inhibitor, Dasatinib (Axon Medchem, Reston, VA, USA) (26). Following tetramer staining, T cells were stained with LIVE/DEAD™ fixable violet dead cell stain (Thermo Fisher, Waltham, Massachusetts, US), and anti-CD8 APC-Vio770 (catalog number 130-113-155, clone BW135/80, Miltenyi Biotec) antibodies. Staining was visualised using a FACS Canto II machine (BD Biosciences, Franklin Lakes, NJ, USA) and subsequently analysed using FlowJo (Tree Star Inc., Ashland, Oregon, US) (V10.7.1). For the tetramer staining of PBMCs and T-cells see below for variations of the tetramer staining methods.

Production of soluble protein

Soluble TCR and pMHC proteins were manufactured as previously described (47). Briefly, codon-optimised synthetic genes for 4C6 TCR α chain, TCR β chain, HLA A*24:02 heavy chain (α1, α2, and α3, domains) and Beta-2-Microglobulin (β2M) chain were generated by Geneart. Sequences were confirmed by Sanger DNA sequencing (Eurofins). TCR expression constructs were designed to contain a non-native disulphide bond, by substituting residues 48 and 57 of TCR α chain and TCR β chain constant domains respectively with cysteine residues, in order to produce stable soluble TCRs (28). The expressed protein sequence is shown in **Supplementary Figure 1.** For production of biotinylated pMHC monomers, a biotin tag (GLNDIFEAQKIEWHE) was added to the C-terminus of the HLA A*24:02 heavy chain construct. The TCR α chain, TCR β chain, the HLA A*24:02 heavy chain and β2M constructs were inserted into separate pGEM-T7 expression plasmids (Promega, Madison, WI, USA) under the control of the T7 promoter. Competent

BL21 *E. coli* cells (Thermo Fisher Scientific) were used to produce proteins in the form of soluble inclusion bodies, using 0.5 mM Isopropyl β-d-1-thiogalactopyranoside (IPTG, Neo Biotech Nanterre, France) to induce expression. Inclusion bodies were chemically refolded to form TCR or pMHC complexes as described previously (47).

Surface plasmon resonance (SPR)

SPR equilibrium binding analysis was performed using a BIAcore T200 (Cytiva, Marlborough, MA, US) equipped with a CM5 sensor chip as previously reported (22, 33). H2-K^d-SIINFEKL was used as a negative control on flow cell 1. SPR kinetic analyses were carried out to determine the K_D values for the TCR, at 25°C. For all kinetic experiments, approximately 500 RUs of biotinylated pMHC was coupled to the CM5 sensor chip surface. Ten TCR samples, made in serial dilution from a starting concentration of 361.5 μM, were sequentially injected over the chip at 30 μL/min. K_D values were calculated assuming 1:1 Langmuir binding [AB=B∗ABMAX/(KD + B)] and the data were analysed using a global fit algorithm (GraphPad Prism). For thermodynamics experiments, the SPR method was repeated at 8, 15, 21, 25, 35, and 40°C. The binding free energies ($\Delta G = RT \ln K_D$) were plotted against temperature and the thermodynamic parameters (ΔH° and TΔS°) were calculated using the non-linear Van't Hoff equation ($RT \ln K_D = \Delta H° - T\Delta S° + \Delta Cp°(T - T°) - T\Delta Cp° \ln (T/T°)$) with T° = 298 K)

Crystal structure determination

HLA A*24:02-QLPRLFPLL and 4C6-HLA A*24:02-QLPRLFPLL protein crystals were grown at 18°C by vapour diffusion via the sitting drop technique as described (40). 200 nL of protein (10 mg/mL) in crystallization buffer (10 mM Tris pH 8.1 and 10 mM NaCl) was added to 200 nL of reservoir solution. HLA A*24:02-QLPRLFPLL crystals were grown in 20% PEG 4000, 0.1 M TRIS (pH 7.5), and 15% glycerol(29). 4C6-HLA A*24:02-QLPRLFPLL crystals were grown in 25% PEG 1500, 0.1 M PCB (48). Crystallization screens were conducted using an Art-Robbins Gryphon dispensing robot (Alpha Biotech Ltd, Killearn, Scotland UK). 4C6-HLA A*24:02-LWMRLLPLL crystals were grown at 18°C by vapour diffusion via hanging drop technique as described (43). 1 μL of protein (10 mg/mL) in crystallisation buffer was added to 1 μL of screen solution (25% PEG 1500, 0.1 M). 4C6-HLA A*24:02-QLPRLFPLL crystals were crushed in 25% PEG 1500, 0.1 M PCB until no visible crystal remained using a MicroBead seed kit (Molecular Dimensions, Sheffield, UK). 0.5 μL of crushed crystal mixture was added to a 25% PEG 1500, 0.1 M PCB solution containing the 4C6 TCR and HLA A*24:02-LWMRLLPLL proteins. X-ray diffraction data were collected at 100 K at the Diamond Light Source (DLS), Oxfordshire, UK at a wavelength of ~0.979 Å using a PILATUS 6M pixel detector. Reflection intensities were estimated using Xia2, XDS and Dials and the data were analysed with the CCP4 package (49). Structures were solved with molecular replacement using PHASER (50). Sequences were adjusted with COOT (51) and the models refined with REFMAC5 (52). Graphical representations were prepared with the PYMOL molecular graphics system, version 2.3.4. The numbering of amino acid residues of the 4C6 TCR was based on the sequences used for bacterial expression as displayed in **Supplementary Figure 1B**. The reflection data and final coordinates were deposited in the PDB database www.rcsb.org (HLA A*24:02-QLPRLFPLL PDB: 7MND; 4C6-HLA A*24:02-LWMRLLPLL

PDB: 7NMG: 4C6-HLA A*24:02-QLPRLFPLL PDB 7NME and 4C6-HLA A*24:02-QLPRLFPLL monoclinic form PDB 7NMF).

Klebsiella oxytoca epitope validation

The 4C6 T-cell clone was rested overnight in R5 media, as above then incubated with antigen presenting cells, with 3 x10^4 4C6 T-cells and 6 x10^4 antigen presenting cells per well of a 96U well plate. The cells were incubated overnight in R5 media at 37°C and 5% CO_2, with all conditions performed in triplicate. Supernatants were harvested and MIP-1β or TNF production measured by enzyme-linked immunosorbent assay (ELISA, R&D Systems, Bio-Techne, Minneapolis, MN, US). Experiment 1 used C1R cells +/- *HLA A*24:02* +/- *IGF2BP2* (irrelevant control), *gsiA* or *fes* genes. Experiment 2 used THP-1 and A549 cells: WT and + *HLA A*A24:02* +/- *gsiA* or *fes* genes. PHA (10 μg/mL) as a positive control and exogenous *Klebsiella* peptides at 10 μM for comparison were also included. MIP-1β or TNF values for 4C6 T-cells or antigen presenting cells incubated alone were subtracted during analysis. Data was analysed in Microsoft Excel then displayed using GraphPad Prism software analysis (v.9.0.1).

Peptide priming of CD8 T-cells

CD8 T-cells from an HLA A*24$^+$ healthy donor (BB51) were magnetically enriched by positive selection using CD8 microbeads according to the manufacturer's instructions (Miltenyi Biotec). CD8 negative cells from the same donor were irradiated then combined with the CD8 T-cells in 24 well plates, with 8 x10^6 and 3 x10^6 cells per well respectively. Cells were cultured in 2 mL of T-cell media per well, recipe as above, but with 20 IU/mL of IL-2 and no IL-15. The media (50%) was changed thrice weekly. For the peptide primed conditions *Klebsiella*-SLPRLFPLL or PPI-LWMRLLPLL peptides were added directly to the wells at a final concentration of 10^{-5} M. The unprimed well received an equivalent volume of DMSO to that of the peptide primed conditions. 2 μg/mL of Ultra-LEAF^{TM} anti-CD28 antibody (catalog number 302934, clone CD28.2, BioLegend) was added at the same time as the peptides or DMSO. After 2 weeks the lines were stained with HLA A*24:02 irrelevant-AYAAAAAAL, *Klebsiella*-SLPRLFPLL and PPI-LWMRLLPLL tetramers.

Magnetic-based pMHC tetramer enrichment from HLA A*24$^+$ patients and healthy donors

For type 1 diabetic patient CCPO-1406, 16.3 x10^6 freshly isolated PBMCs were treated with 50 nM PKI (26) for 30 min at 37°C then labelled with 0.5 μg PE conjugated PPI-LWMRLLPLL tetramer per 3 x10^6 cells for 30 min on ice. Excess tetramer was washed from the cells with ice-cold MACS buffer, then tetramer labelled cells enriched using anti-PE magnetic microbeads according to the manufacturer's instructions (Miltenyi Biotec) and as previously described (14). Positive cells from the column were incubated overnight in a well of a 96U plate then expanded the next day *in situ* with mixed irradiated (3100 cGy) PBMCs from three donors and 1 μg/mL of PHA. As the cells expanded, they were divided between 96U wells then transferred to wells of a 48 well plate and kept at a 0.5-1 x10^6 per well. The cells were re-stained with HLA A*A24 *Klebsiella*-SLPRLFPLL and PPI-LWMRLLPLL tetramers, using PKI pre-treatment and tetramer staining stabilisation with an anti-PE unconjugated antibody (catalog number 408102, clone PE001, BioLegend) (53).

For type 1 diabetic patient T1D-12, 1 x10^6 freshly isolated PBMCs were initially amplified for 2 weeks in T-cell media with CD3/CD28 Dynabeads according to the manufacturer's instructions (ThermoFisher Scientific), followed by 42 x10^6 of the expanded T-cells undergoing enrichment with PE conjugated PPI-LWMRLLPLL tetramer, then expansion and staining with tetramers as described above.

For the healthy donors, cryopreserved PBMCs (30.1-90.6 x10^6 cells, mean 55.9 x10^6) were defrosted and used for tetramer enrichment. Whereas T-cells from the type 1 diabetic donors were enriched with PPI-LWMRLLPLL tetramer, the healthy donors were enriched with either *Klebsiella oxytoca*-SLPRLFPLL or HLA A*24:02 CMV-AYAQKIFKIL tetramers, using the same approach as described above for CCPO-1406. Once expanded the T-cells enriched with *Klebsiella oxytoca*-SLPRLFPLL were re-stained with HLA A*A24 irrelevant-AYAAAAAAL, *Klebsiella*-SLPRLFPLL, *Klebsiella*-RYPRLFGIV and PPI-LWMRLLPLL tetramers. The CMV enriched lines were stained with irrelevant-AYAAAAAAL, CMV-AYAQKIFKIL and PPI-LWMRLLPLL tetramers.

The process of magnetic enrichment with tetramers followed by expansion was performed once (T1D-12, BB51, BB52, 572D) or twice (CCPO-1406, BB31, BB25, BB64, BB57), depending on the number of cells available and percentage of tetramer staining seen after the first enrichment.

Flow cytometry-based pMHC tetramer sorting and TCR sequencing

T-cells lines were pre-treated with the PKI Dasatinib (26), then stained with PE and APC conjugated tetramers assembled with the same epitope: SLPRLFPLL-PE versus SLPRLFPLL-APC, or LWMRLLPLL-PE versus LWMRLLPLL-APC. The tetramers were pre-mixed immediately before adding to the cells with 0.25 µg of each tetramer used per 0.2 x10^6 of cells. Anti-PE (as above) and anti-APC (catalog number 408002, clone APC003, BioLegend) unconjugated antibodies were used to stabilise tetramer staining. LIVE/DEAD™ fixable violet dead cell stain, CD3-PerCP (catalog number 130-113-131, clone BW264/56, Miltenyi Biotec) and CD8-FITC (catalog number 130-110-677, clone REA734, Miltenyi Biotec) were used to stain the cells post tetramer staining. Sorting was performed on a BD FACS Aria III (BD Biosciences) by Central Biotechnology Services at Cardiff University and gated as follows: lymphocytes (FSC-A versus SSC-A), single cells (FSC-A versus FSC-H), viable (LIVE/DEAD™ fixable violet dead cell stain low/negative) $CD3^+$ cells, $CD8^+$ cells, then PE versus APC tetramer. Irrelevant AYAAAAAAL-PE versus AYAAAAAAL-APC tetramer staining was used to set the sorting gate. $Tetramer^+$ cells were collected directly into 300 µL of RNAprotect cell reagent (Qiagen, Hilden, Germany) according to the manufacturer's instructions, then used for TCR sequencing. RNA extraction was carried out using the RNeasy Micro kit (Qiagen). cDNA was synthesized using the 5′/3′ SMARTer kit according to the manufacturer's instructions (Takara Bio, Paris, France). The SMARTer approach used a Murine Moloney Leukaemia Virus (MMLV) reverse transcriptase, a 3′ oligo-dT primer and a 5′ oligonucleotide to generate cDNA templates flanked by a known, universal anchor sequence at the 5′. A Step-Out PCR was performed using a pair of primers consisting of 3' TRAC or TRBC-specific reverse primer (Eurofins Genomics) and a 5' universal anchor-

specific forward primer (Takara Bio). All samples were used for the following PCR reaction: 2.5 µL template cDNA, 0. 5 µL High Fidelity Phusion Taq polymerase, 10 µL 5X Phusion buffer, 0.5 µL DMSO, 1 µL dNTP Mix (stock concentration of 10 mM of each) (all from Thermo Fisher Scientific), 1 µL of TRAC or TRBC-specific primer (10 µM stock), 5 µL of 10X anchor-specific universal primer (Takara Bio), and nuclease-free water for a final reaction volume of 50 µL (cycling conditions: 5 min at 94°C, 30 cycles of 30 s at 94°C, 30 s at 63°C for alpha chains or 30 s at 66°C for beta chains, 120 s at 72°C). Subsequently, 5 µL of the Step-out PCR products were taken for a nested PCR, using 1 µL of barcoded forward (universal) and reverse (TRAC or TRBC) primers (10 µM stock) (Eurofins Genomics), 0.5 µL High Fidelity Phusion Taq polymerase, 10 µL 5X Phusion buffer, 0.5 µL DMSO, 1 µL dNTP Mix (stock concentration of 10 mM each) and nuclease-free water for a final reaction volume of 50 µL (cycling conditions: 5 min at 94°C, 30 cycles of 30 s at 94°C, 30 s at 62°C, 120 s at 72°C, and a final 10 min at 72°C). The final PCR products were loaded on a 1% agarose gel and purified with the Monarch® gel extraction kit (New England Biolabs, Ipswich, MA, US). Purified products were sequenced on an Illumina MiSeq instrument using the MiSeq v2 reagent kit (Illumina, Cambridge, UK) according to the manufacturer's instructions. Sequence analysis was performed using MiXCR software (v3.0.7) (54). TCRs appearing in both SLPRLFPLL and LWMRLLPLL tetramer sorted populations were deemed as crossreactive.

Flow cytometry-based T-cell activation assays

T-cells were rested in R5 (as for R10 with 5% FBS) for 24 h before setting up the assay to help reduce spontaneous activation. For TNF-processing inhibitor (TAPI)-0 (Santa Cruz Biotechnology TX, US) assays, T-cells (3 x10^4) and target (6 x10^4) cells were co-incubated for 4-6 h with 30 µM TAPI-0 (Merck), and with antibodies directed against TNF (catalog number 130-120-492, clone cA2, PE-Vio770 conjugated, Miltenyi Biotec) and CD107a (clone H4A3, FITC conjugated catalog number 555800, or PE conjugated catalog number 555801, BD Biosciences), with the latter detecting activation-induced degranulation of cytotoxic T-cells. Following co-incubation, cells were washed then stained with LIVE/DEAD™ fixable violet dead cell stain for 5 min at RT, FcR blocking reagent used according to the manufacturer's instructions (Miltenyi Biotec), and without washing antibodies against CD3 PerCP (as above) and CD8 APC (as above) added and incubated for 20 min on ice. Cells were washed in PBS and acquired immediately on a flow cytometer or fixed with 2% formaldehyde for acquisition within 24 h. Acquisition was performed on a ACEA NovoCyte 3005 with NovoSampler pro (ACEA, Agilent, Santa Clara, CA, USA). Gating was for lymphocytes (FSC-A/H versus SSC-A/H), single cells (FSC-A versus FSC-H), viable $CD3^+$ cells (LIVE/DEAD™ fixable violet dead cell stain low/negative), $CD3^+$/$CD8^+$, then displayed as TNF versus CD107a.

Cytotoxicity assays

Chromium 51 (^{51}Cr) assays were performed using K-562 cells expressing HLA A*24:02 with and without preproinsulin as target cells for T-cell lines from donor BB57. K-562 cells were labelled with ^{51}Cr as sodium chromate (Perkin Elmer, Waltham, MA) and incubated at 37°C for 1 h then washed in R10 medium and incubated in 2 mL R10 medium for a further hour to allow excess ^{51}Cr to leach from the cells. Target cells (2,000 per well) were incubated with T-cell lines to give desired T-cell to K-562 cell ratios. Targets were

incubated alone or with lysis buffer (5% Triton in H_2O) to give spontaneous and maximum release of ^{51}Cr respectively. After incubation (6 h), culture supernatant was harvested and mixed with 150 µL of Opitphase Supermix scintillation cocktail (PerkinElmer) and the release of ^{51}Cr measured using a 1450 MicroBeta TriLux (PerkinElmer). Percentage killing was determined using the following calculation:

$$\%\ \text{killing}\ = 100 - \left(\left(\frac{\text{release from K-562 with T-cells} - \text{spontaneous release from K-562}}{\text{total release from K-562} - \text{spontaneous release from K-562}}\right) \times 100\right)$$

Supplementary References

The references above conform with the manuscript reference numbers but include the additional ten references listed below:

43. Galloway SAE, Dolton G, Attaf M, Wall A, Fuller A, Rius C, et al. Peptide Super-Agonist Enhances T-Cell Responses to Melanoma. *Frontiers in immunology.* 2019;10:319.

44. Theaker SM, Rius C, Greenshields-Watson A, Lloyd A, Trimby A, Fuller A, et al. T-cell libraries allow simple parallel generation of multiple peptide-specific human T-cell clones. *J Immunol Methods.* 2016;430:43-50.

45. Dolton G, Rius C, Wall A, Szomolay B, Bianchi V, Galloway SAE, et al. Targeting of multiple tumor-associated antigens by individual T cell receptors during successful cancer immunotherapy. *Cell.* 2023;186(16):3333-49 e27.

46. Wooldridge L, Lissina A, Cole DK, van den Berg HA, Price DA, and Sewell AK. Tricks with tetramers: how to get the most from multimeric peptide-MHC. *Immunology.* 2009;126(2):147-64.

47. Boulter JM, Glick M, Todorov PT, Baston E, Sami M, Rizkallah P, et al. Stable, soluble T-cell receptor molecules for crystallization and therapeutics. *Protein Eng.* 2003;16(9):707-11.

48. Newman J, Egan D, Walter TS, Meged R, Berry I, Ben Jelloul M, et al. Towards rationalization of crystallization screening for small- to medium-sized academic laboratories: the PACT/JCSG+ strategy. *Acta Crystallogr D Biol Crystallogr.* 2005;61(Pt 10):1426-31.

49. Winn MD, Ballard CC, Cowtan KD, Dodson EJ, Emsley P, Evans PR, et al. Overview of the CCP4 suite and current developments. *Acta Crystallogr D Biol Crystallogr.* 2011;67(Pt 4):235-42.

50. McCoy AJ, Grosse-Kunstleve RW, Adams PD, Winn MD, Storoni LC, and Read RJ. Phaser crystallographic software. *Journal of applied crystallography.* 2007;40(Pt 4):658-74.

51. Emsley P, and Cowtan K. Coot: model-building tools for molecular graphics. *Acta Crystallogr D Biol Crystallogr.* 2004;60(Pt 12 Pt 1):2126-32.

52. Murshudov GN, Skubak P, Lebedev AA, Pannu NS, Steiner RA, Nicholls RA, et al. REFMAC5 for the refinement of macromolecular crystal structures. *Acta Crystallogr D Biol Crystallogr.* 2011;67(Pt 4):355-67.

53. Tungatt K, Bianchi V, Crowther MD, Powell WE, Schauenburg AJ, Trimby A, et al. Antibody stabilization of peptide-MHC multimers reveals functional T cells bearing extremely low-affinity TCRs. *J Immunol.* 2015;194(1):463-74.

54. Bolotin, D.A. *et al.* MiXCR: software for comprehensive adaptive immunity profiling. *Nature methods* **12**, 380-381 (2015).

A

4C6α native sequence: leader, TRAV05*01, CDR3α, TRAJ37*01 and TRAC1:

atgaagacatttgctggattttcgttcctgtttttgtggctgcagctggactgtatgagtagaggagaggatgtggag
cagagtcttttcctgagtgtccgagagggagacagctccgttataaactgcacttacacagacagctcctccacctac
ttatactggtataagcaagaacctggagcaggtctccagttgctgacgtatatttttcaaatatggacatgaaacaa
gaccaaagactcactgttctattgaataaaaaggataaacatctgtctctgcgcattgcagacacccagactggggac
tcagctatctacttc**tgcgcggaaccgagcggcaacaccggcaaactgatttttt**gggcaagggacaactttacaagta
aaaccagatatccagaaccctgaccctgccgtgtaccagctgagagactctaaatccagtgacaagtctgtctgccta
ttcaccgattttgattctcaaacaaatgtgtcacaaagtaaggattctgatgtgtatatcacagacaaaactgtgcta
gacatgaggtctatggacttcaagagcaacagtgctgtggcctggagcaacaaatctgactttgcatgtgcaaacgcc
ttcaacaacagcattattccagaagacaccttcttccccagcccagaaagttcctgtgatgtcaagctggtcgagaaa
agctttgaaacagatacgaacctaaactttcaaaacctgtcagtgattgggttccgaatcctcctcctgaaagtggcc
gggtttaatctgctcatgacgctgcggctgtggtccagctga

4C6β native sequence: leader, TRBV7-9*03, CDR3β, TRBJ2-7*01 and TRBC2*01:

atgggcaccagcctcctctgctggatggccctgtgtctcctgggggcagatcacgcagatactggagtctcccaggac
cccagacacaagatcacaaagaggggacagaatgtaactttcaggtgtgatccaatttctgaacacaaccgcctttat
tggtaccgacagaccctggggcagggcccagagtttctgacttacttccagaatgaagctcaactagaaaaatcaagg
ctgctcagtgatcggttctctgcagagaggcctaagggatctttctccaccttggagatccagcgcacagagcagggg
gactcggccatgtatctc**tgcgcgagcagcctgcatcatgaacagtatttt**gggccgggcaccaggctcacggtcaca
gaggacctgaaaaacgtgttcccacccgaggtcgctgtgtttgagccatcagaagcagagatctcccacacccaaaag
gccacactggtgtgcctggccacaggcttctaccccgaccacgtggagctgagctggtgggtgaatggaaggaggtg
cacagtggggtcagcacagacccgcagcccctcaaggagcagcccgccctcaatgactccagatactgcctgagcagc
cgcctgagggtctcggccaccttctggcagaacccccgcaaccacttccgctgtcaagtccagttctacgggctctcg
gagaatgacgagtggacccaggatagggccaaacctgtcacccagatcgtcagcgccgaggcctggggtagagcagac
tgtggcttcacctccgagtcttaccagcaaggggtcctgtctgccaccatcctctatgagatcttgctagggaaggcc
accttgtatgccgtgctggtcagtgccctcgtgctgatggccatggtcaagagaaaggattccagaggc

B

4C6α protein construct: V-domain, CDR1α, CDR2α, CDR3α, C-domain, non-native cysteine and stop codons (**):

MGEDVEQSLFLSVREGDSSVINCTYT**DSSSTY**LYWYKQEPGAGLQLLTY**IFSNMDM**KQDQRLTVLLN
KKDKHLSLRIADTQTGDSAIYF**CAEPSGNTGKLIF**GQGTTLQVKPIQNPDPAVYQLRDSKSSDKSVC
LFTDFDSQTNVSQSKDSDVYITDK**C**VLDMRSMDFKSNSAVAWSNKSDFACANAFNNSIIPEDTFFPS
PESS**

4C6β protein construct: V-domain, CDR1β, CDR2β, CDR3β, C-domain, non-native cysteine and stop codons (**):

MDTGVSQDPRHKITKRGQNVTFRCDPI**SEHNR**LYWYRQTLGQGPEFLTY**FQNEAQ**LEKSRLLSDRFS
AERPKGSFSTLEIQRTEQGDSAMYL**CASSLHHEQYF**GPGTRLTVTEDLKNVFPPEVAVFEPSEAEIS
HTQKATLVCLATGFYPDHVELSWWVNGKEVHSGV**C**TDPQPLKEQPALNDSRY**A**LSSRLRVSATFWQ**D**
PRNHFRCQVQFYGLSENDEWTQDRAKPVTQIVSAEAWGRAD**

Supplementary Figure 1: Sequence of the 4C6 TCR. (A) Native nucleotide sequence of TCRα and TCRβ genes showing VJ assignment (unequivocal assignment of TRBD gene not possible). (**B**) Bacterially expressed protein sequences manufactured in *E.coli* and refolded to make soluble 4C6 TCR for biophysical and structural studies. Sequences include non-native cysteine residues as indicated in bold underlined red text to form non-native disulphide bonding between the TCR a and b constant domains. Two other substitutions in the TCRβ chain that aid refolding are indicated in bold underlined text. The Cys to Ala substitution was included to remove the possibility of incorrect disulphide bind formation.

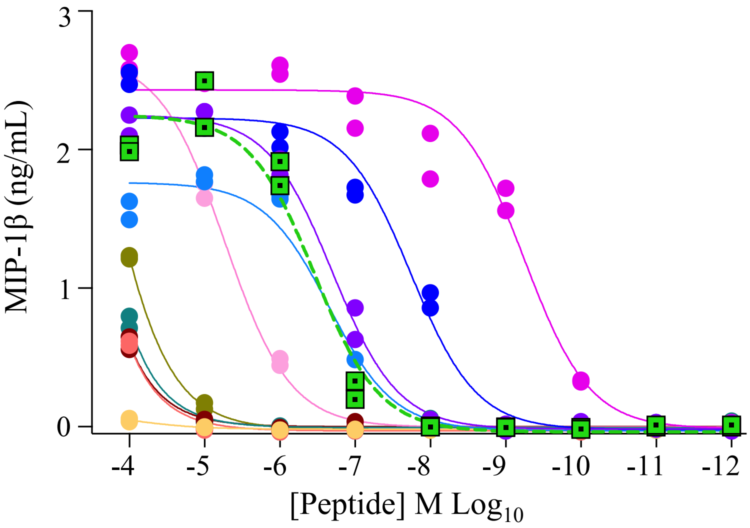

Score	Sequence	EC_{50}
-15.83	LWMRLLPLL	3.49×10^{-7}
-9.97	QWPRLFPIV	**2.00×10^{-7}**
-10.48	NMPRLFPIV	**1.82×10^{-8}**
-10.59	NYLRLAPIL	**2.23×10^{-7}**
-10.67	NLPPLFPIW	NC
-10.70	QLPRLFPLL	**5.66×10^{-10}**
-10.87	QLLRLAGIV	~ 0.74
-10.96	DLLRLAPIL	~ 0.52
-11.03	NYPPLAGIV	5.41×10^{-6}
-11.22	DLMRLAPIW	2.55×10^{-4}
-11.37	HMPRLAPIV	~ 0.2173

Supplementary Figure 2: Identification of a superagonist peptide for 4C6 T-cells. Top ten scoring peptides from 500 'randomly' selected peptides based on the PS-CPL of 4C6, tested in comparison to the preproinsulin peptide LWMRLLPLL. Incubation overnight with C1Rs expressing HLA A24:02 as antigen presenting cells. Crude (>40%) purity peptides used for screening. Assay supernatants harvested for MIP-1β ELISA. Data points shown for duplicate conditions. EC_{50} values in bold indicated peptides seen more sensitively than the preproinsulin peptide. NC = EC_{50} not calculated.

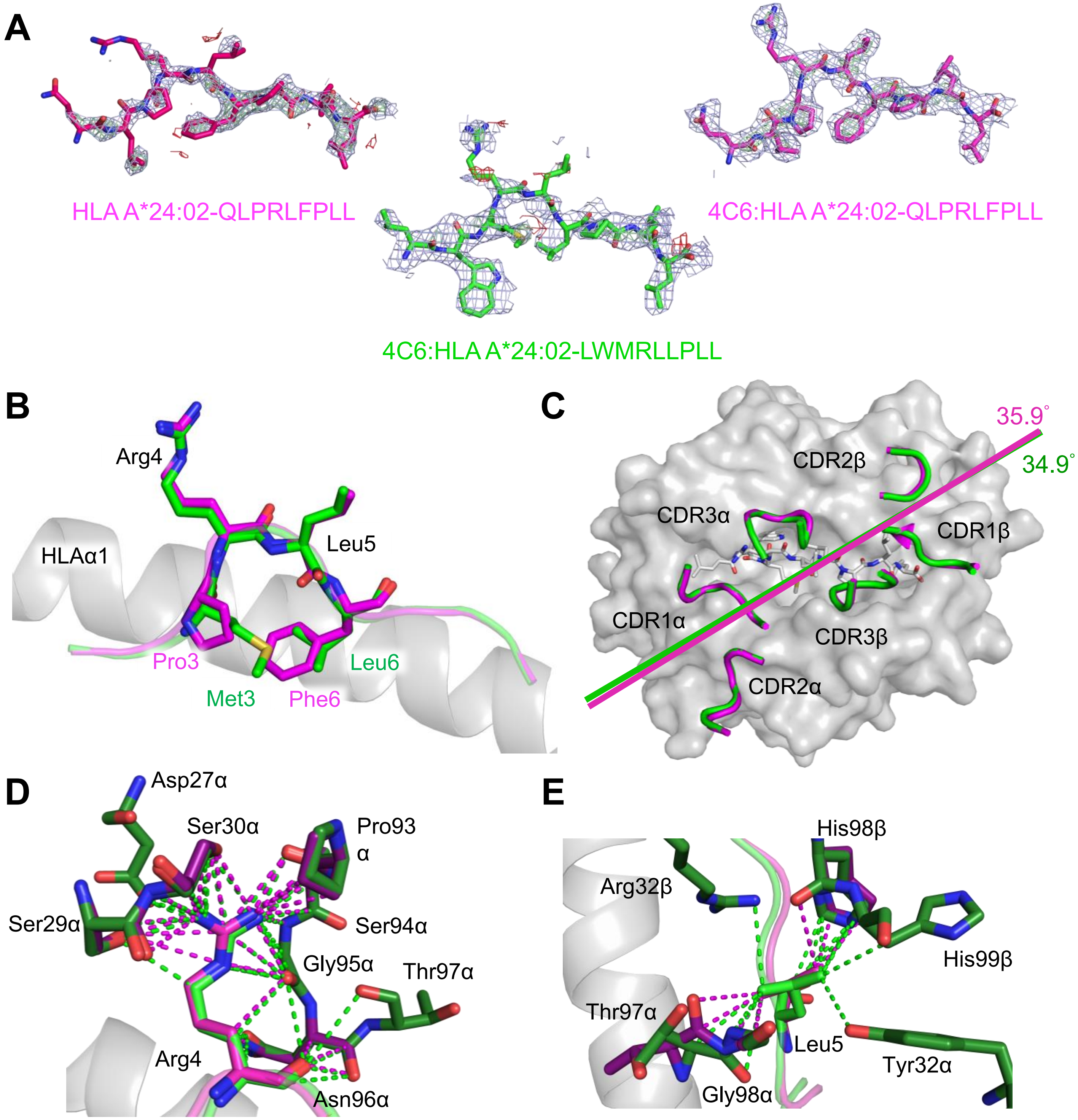

Supplementary Figure 3. Molecular comparison of 4C6:HLA A*24:02-LWMRLLPLL and 4C6:HLA A*24:02-QLPRLFPLL complexes. (A) Electron Density maps of HLA A*24:02-QLPRLFPLL, 4C6:HLA A*24:02-LWMRLLPLL, 4C6:HLA A*24:02-QLPRLFPLL (left to right) structures **(B)** LWMRLLPLL (green) and QLPRLFPPL (magenta) peptides shown as sticks with MHC alpha-helix (grey) shown for orientation. **(C)** Top-down view of 4C6 binding footprint on HLA A24:02 presenting LWMRLLPLL (green) and QLPRLFPPL (magenta) peptides. Peptides shown as white sticks. Green and magenta lines and numbers indicate crossing angles of LWMRLLPLL interaction and QLPRLFPPL interaction respectively **(D-E)** LWMRLLPLL and QLPRLFPLL peptide residues Arg4 **(D)** and Leu5 **(E)** shown as green and magenta sticks respectively. Important 4C6 TCR residues bound to LWMRLLPLL and QLPRLFPLL are shown as dark green and dark purple respectively. Dotted lines indicate interactions between the 4C6 TCR and LWMRLLPLL (green) or QLPRLFPLL (magenta).

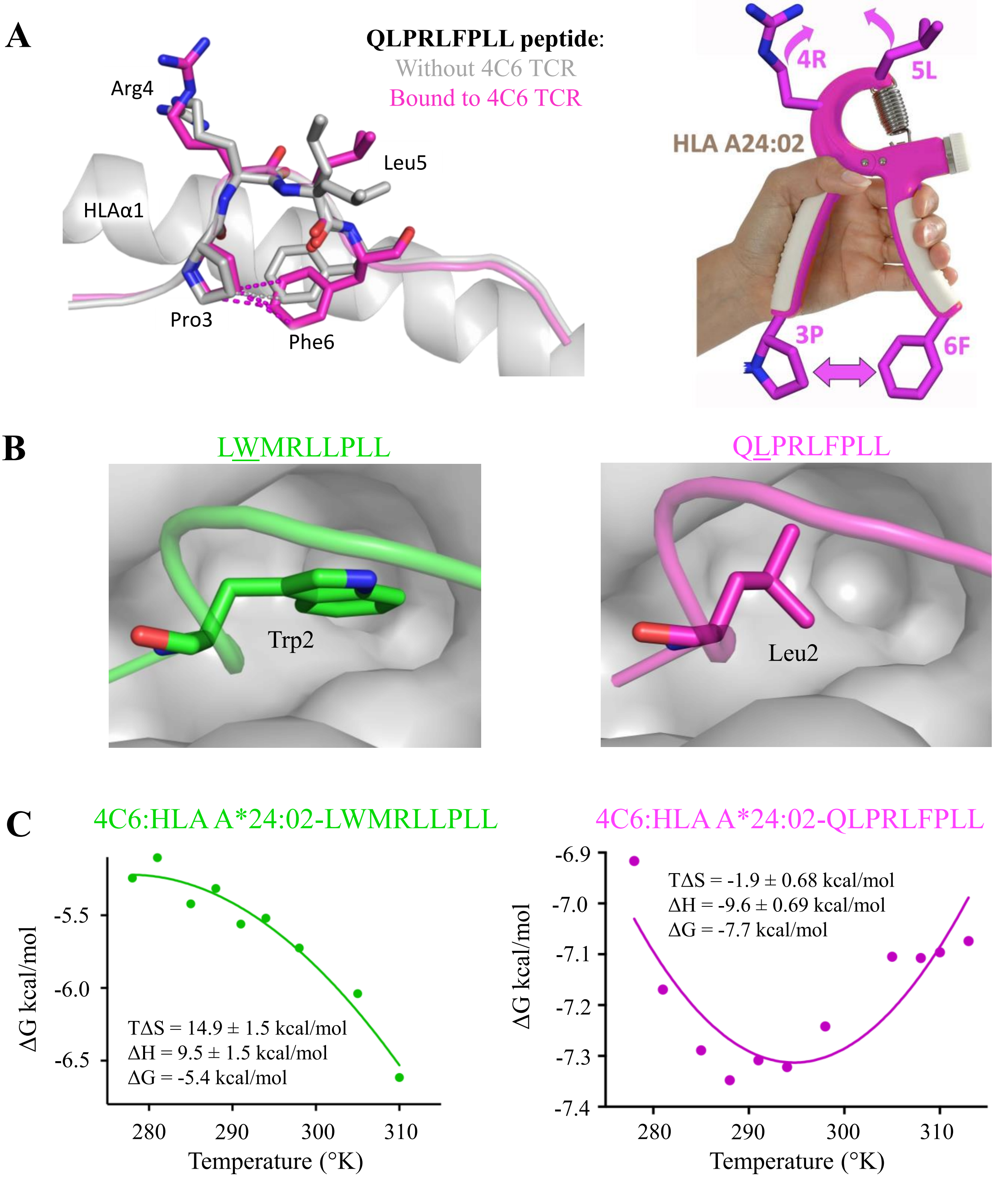

Supplementary Figure 4: Further comparison of 4C6:HLA A*24:02-LWMRLLPLL and 4C6:HLA A*24:02-QLPRLFPLL complexes. A. Strong intra-peptide interaction between Pro3 and Phe6 acts to stabilise and position the central Arg4/Leu5 bulge in the HLA A*24:02 binding groove. Pro3 and Phe6 form 1 VdW interaction (<4 Å) in the unbound structure and 3 interactions when complexed to the 4C6 TCR. **B**. Leu2 in QLPRLFPLL acts as a suboptimal anchor and does not fill the HLA A*24:02 B-pocket. **C**. Thermodynamic analysis of 4C6:HLA A*24:02-LWMRLLPLL (green) and 4C6:HLA A*24:02-QLPRLFPLL conducted using SPR. The binding free energies, ΔG (ΔG = RTlnKD), were plotted against temperature (K) using non- linear regression to fit the three-parameters van 't Hoff equation, (RT ln KD = ΔH° –TΔS° + ΔCp°(T-T0) – TΔCp° ln (T/T0) with T0=298 K) to find ΔS and ΔH. Gibbs Free Energy Equation (ΔG = TΔS- ΔH, T = 298K) was used to determine final ΔG value.

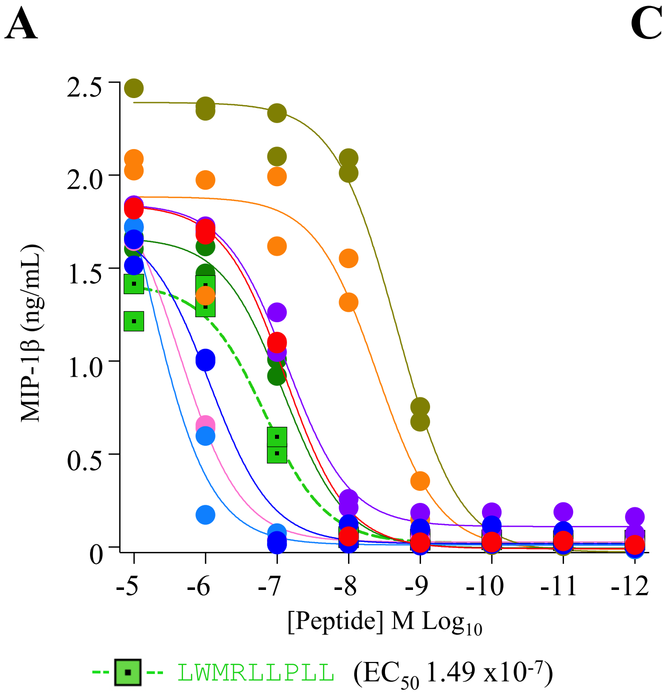

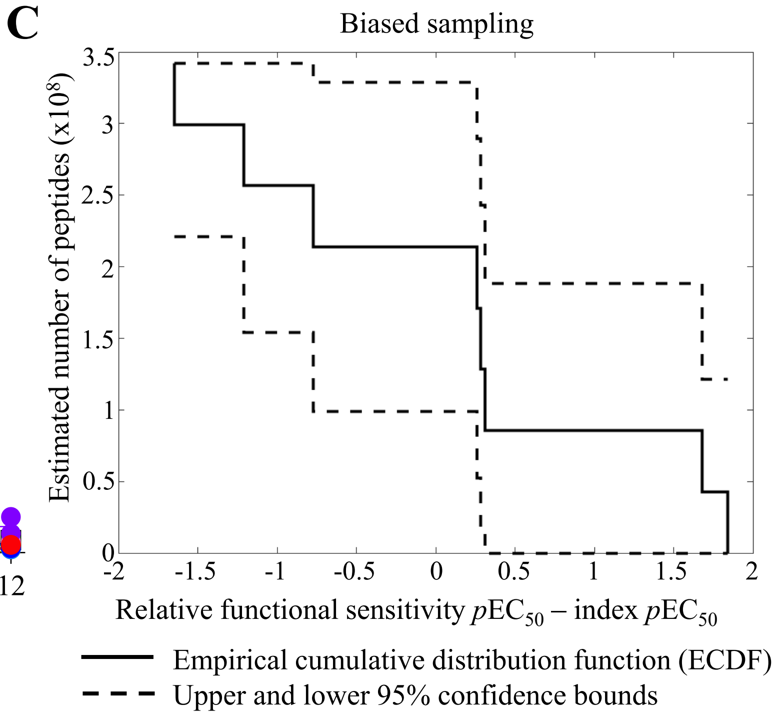

B

Sampling probability	Peptide	EC_{50}	Sampling probability	Peptide	EC_{50}	Sampling probability	Peptide	EC_{50}
0.08510	AHPPLFNVM	NR	0.00946	QHPPLASLI	NR	0.00444	DMMPLLPII	NR
0.07841	RLLPLVSVI	NR	0.00910	QKLPLLGLL	NR	0.00440	EWQPLFSIA	NR
0.04296	ILMPLMPMT	NR	0.00874	EMQPLFGMV	NR	0.00436	HYQPLVPVL	NR
0.04268	DLQRLVSVI	NR	0.00825	QMQRLVNIV	NR	0.00432	SNPPLMGLM	NR
0.03295	WNLPLAGMI	~0.08	0.00698	*NNMPLMPMW	7.29×10^{-8}	0.00421	RYPPLVPML	NR
0.03223	SWLRLFNMM	NR	0.00668	LMMPLAPMM	NR	0.00407	LWLRLAGMM	6.62×10^{-6}
0.02593	ENMPLMPMI	NR	0.00667	IWQPLAGVA	NR	0.00405	SLLPLLGIL	NR
0.02446	LYQPLVGMV	NR	0.00664	QYLRLFNIT	NR	0.00400	*KYPRLMNIV	2.13×10^{-9}
0.02230	QNPRLMSVM	~0.07	0.00627	*KYPRLMSLM	8.15×10^{-8}	0.00385	KNLPLVPII	NR
0.02001	EYPPLLNIL	NR	0.00613	EYMPLVGML	NR	0.00367	NMLRLMNIM	~0.07
0.01908	SMMRLLPVW	NR	0.00563	SNMRLMPVV	NR	0.00353	RLMPLVPVV	NR
0.01735	ELPPLMSVW	NR	0.00562	ALMRLFSLF	NR	0.00345	RMQRLVPLA	2.38×10^{-6}
0.01503	IWPPLVPVT	NR	0.00530	QMPPLVNIM	NR	0.00341	NHMPLFSIA	~0.05
0.01472	*QYPRLLNLV	7.73×10^{-8}	0.00520	NKLRLVNIM	NR	0.00336	LLMPLVGLW	NR
0.01409	QWPRLVNVF	NR	0.00501	QMLPLLPLF	NR	0.00323	QKQRLASIL	NR
0.01249	QKQPLLPII	NR	0.00487	KWLRLMPMM	NR	0.00318	DHPPLFPMA	NR
0.01224	RHMRLFGML	NR	0.00487	NLMRLMNLA	NR	0.00301	DKPRLVPVI	NR
0.01213	LKMRLLGIA	8.77×10^{-7}	0.00486	NHPRLVGVI	NR	0.00299	DNPRLFGLT	NR
0.01208	DYQRLVNLL	NR	0.00474	SYPPLVGMV	NR	0.00284	*EYQRLMPVL	3.11×10^{-9}
0.00954	HYMPLFGVT	NR	0.00457	NWQPLVSIW	NR	0.00268	RNPPLVGIW	NR

Supplementary Figure 5: Estimate of the number of 9mer peptides 4C6 can recognize in the context of HLA A*24:02 by biased sampling. (**A** and **B**) Raw data from the primary PS-CPL of 4C6 was modified by excluding cysteine, then normalized to provide peptide sampling distribution to bias the sample toward good agonists. A total of 60 peptides were drawn from an effective size of 3.16×10^6 from the sampling entropy. (**A**) 4C6 T-cells were incubated overnight with peptide and C1R-HLA A24:02 as antigen presenting cells. Assay supernatants used for MIP-1β ELISA. Data points shown for duplicate conditions. (**B**) The 60 peptides and EC_{50} of 4C6 activation. Eight of the peptides were recognized with a $pEC_{50}>5$ (highlighted in color) and 5 (*) were better agonists than the index peptide. (**C**) Plotted ECDF with lower and upper 95% confidence bounds using MATLAB 2019a.

A

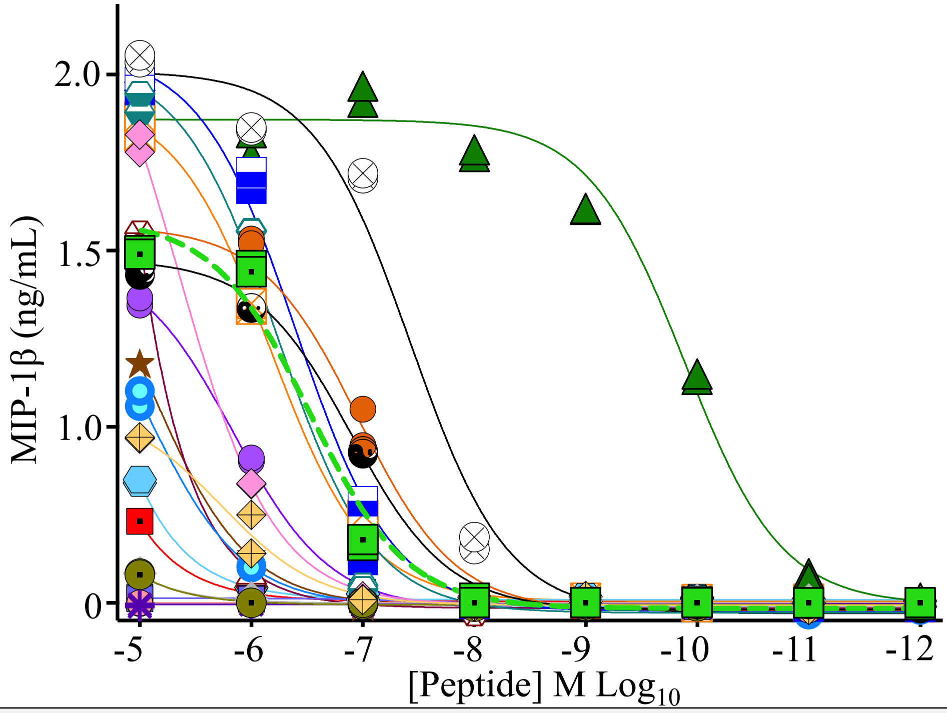

B

Score	Peptide	EC_{50}	Organism	Protein
-15.83	LWMRLLPLL	2.7 x10^{-7}	*Homo sapiens*	Preproinsulin
-9.63	QLPPLFPIV	**1.4 x10^{-7}**	*Aspergillus oryzae*	Ubiquitin-like activating enzyme
-10.23	SYPPLAPIV	3.2 x10^{-6}	*Fusarium oxysporum*	Putative protein
-10.53	NLLPLAPLA	~ 0.03	*Cryptococcus neoformans*	Nucleoside-diphosphatase putative
-10.65	QYPPLVPIM	**2.1 x10^{-8}**	*Aspergillus oryzae*	Unnamed protein product
-10.70	HLPRLAGLV	~ 0.003	*Fusarium oxysporum*	Putative protein
-10.92	EYPPLVPIL	NR	*Aspergillus niger*	Unnamed protein product
-10.92	ALPPLFPIA	**1.3 x10^{-7}**	*Aspergillus oryzae*	Putative protein
-11.05	NLPPLAPIT	4.5 x10^{-6}	*Aspergillus oryzae*	Dicer-like protein 2
-11.21	NLLRLVGLV	5.6 x10^{-7}	*Aspergillus niger*	Putative protein
-11.24	EYLPLFPLL	**5.2 x10^{-8}**	*Fusarium oxysporum*	Putative protein
-11.27	SLMRLFPLL	**3.8 x10^{-8}**	*Fusarium oxysporum*	Putative protein
-11.39	ALQRLAPIL	4.4 x10^{-7}	*Fusarium oxysporum*	Putative protein
-11.41	ILQRLAPIL	7.5 x10^{-6}	*Fusarium oxysporum*	Putative protein
-11.53	KKPRLAGIL	~ 0.08	*Sporothrix schenckii*	Putative protein
-11.75	LLLPLAGIL	1.2 x10^{-6}	*Fusarium oxysporum*	Putative protein
-12.01	DMPRLAGVL	6.4 x10^{-6}	*Sporothrix schenckii*	Putative protein
-12.04	LLPRLFGLF	**1.3 x10^{-10}**	*Cryptococcus neoformans*	Cytoplasmic protein
-12.10	RLPPLAPLI	~ 0.02	*Cryptococcus neoformans*	NAD+ diphosphatase
-12.18	LNPRLFGIL	6.8 x10^{-7}	*Fusarium oxysporum*	Delta24(24(1))-sterol reductase
-12.26	ALMRLMGIA	~ 0.06	*Saccharomyces cerevisiae*	Putative protein

Supplementary Figure 6: 4C6 cross-reacts with fungus derived peptides. Positional-scanning combinatorial peptide library data for 4C6 was used to screen a database of disease fungal species and the top 20 peptides selected for testing. (**A**) Peptide titrations using 4C6 and the top 20 fungal peptides (listed in B). Incubation overnight with C1Rs expressing HLA A*24:02 as antigen presenting cells. Assay supernatants used for MIP-1β ELISA. Data points shown for duplicate conditions. (**B**) Peptide sequence and origin. Scoring indicates prediction of how likely the peptide is to be recognized by 4C6 T-cells, with the best scoring peptide at the top. EC_{50} of activation in bold indicate peptides seen more sensitively than the preproinsulin peptide in functional assays.

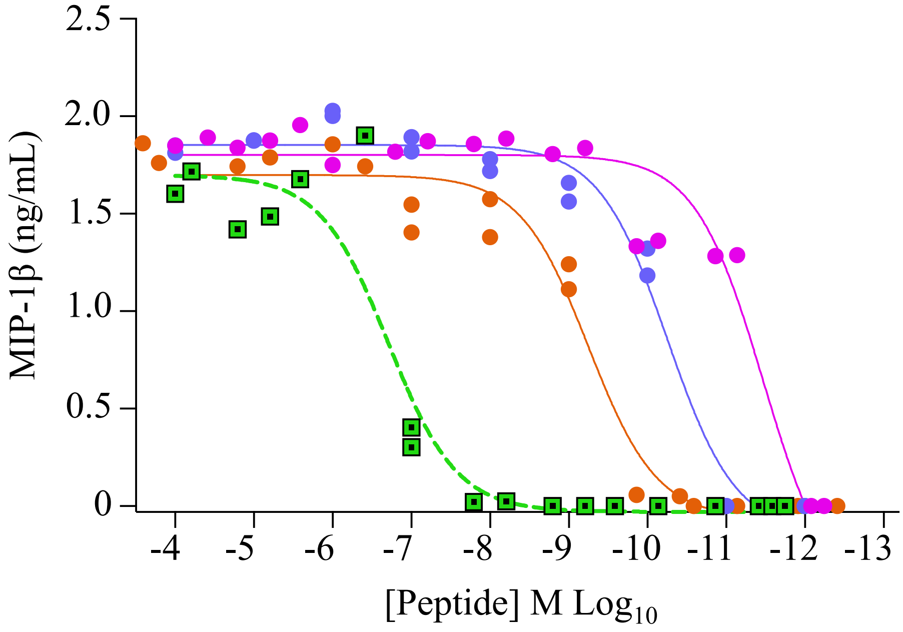

Score	Sequence	EC_{50}	Origin
-15.83	LWM**RL**L**PLL**	1.9×10^{-7}	Preproinsulin
-9.97	**QLPRLFPLL**	3.4×10^{-12}	Superagonist screen
-10.48	S**LPRLFPLL**	5.6×10^{-11}	*Klebsiella oxytoca*
-10.59	RY**PRLF**GIV	5.6×10^{-10}	*Klebsiella oxytoca*

Supplementary Figure 7: Comparative sensitivity of 4C6 T-cells to preproinsulin, superagonist and *Klebsiella oxytoca* peptides. Sensitivity of 4C6 T-cells to preproinsulin (LWMRLLPLL), super-agonist (QLPRLFPLL) and *Klebsiella oxytoca* (SLPRLFPLL and RYPRLFGIV) peptides in a titration assay. Underlined amino acid residues are the same as the preproinsulin peptide. Residues in bold are present in the superagonist. Incubation overnight with C1R-HLA A*24:02 as antigen presenting cells. Assay supernatants used for MIP-1β ELISA. Data points shown for duplicate conditions.

A Enterochelin esterase (DUF3327 containing protein): *fes* gene

MKTAWSGLLIGMSTLPAAAMNCESPSLQGELEGKFDASGEVCFDLPTLGENYVAATLSGVTDARLLDDNNRRLRTL
LTGGPADGEQTLLFSLPVNRASSLVLHGEEGARWRFQWQMRETTALAKIQPLDPVSPRLLRLKRELAAGGSTAHFW
QEREREGTPMVEPVDAGHKRVTFLWRGASRNVFILGSPAGDHDPLFRLGDSDVWFRSYVVPADTLMQYKLAPDVPQ
VTGSAREQRRAILVSAQADPLNPNSINAAHDRWNRHSLLALNAVRFCTTQRMAQPLRYGTLTRHQLRSDFLQNTRE
VLIYRPRLPQPARWTLLLFDGKTYQHEYRFANVLDALIASHRLPPINVVFIDSLDHPRRAKELPPNPHFADFMAHE
LLPWLNRQGLSLSRQKTVVAGSSYGGLAASWVAL**RYPRLFGIV**LSLSGSYWWAPQGEEAGWLTRQYQQSPRYPVRF
LLQAGKFETSGPDGGIYANTLEFERVLKEKGYTVSFHPSSSGHDYAAWCEALVNGMRDLTGLALKGKPATPDPAQQ
QIFNRAQR

Glutathione ABC transporter ATP-binding protein: *gsiA* gene

MPHSQEIDADDVLQVRGLNVVFNQQQQAFSAVRDLSFTLRRGETLAIVGESGSGKSVTSLALMRLLDQAVSEVRSD
ALLLRRRSRQVIELSEQSETEMRGVRGADMAMIFQEPMTSLNPVFTIGEQIAESIRLHQGLKHDEALREAKKMLDQV
RIPEAEAMLSRYPHQLSGGMRQRVMIAMALSCRPAVLIADEPTTALDVTIQAQILQLIAVLQKEMAMGVIFITHDMG
VVADIADRVLVMYRGEAVETGSVEEIFRAPKHPYTQALLAAVPRLGAMRGT**SLPRLFPLL**NQSPSPEGQNTVVPGEP
ILKVRDLVARFPLRSGILNRITREVHAVEKVSFDLWPGETLSLVGESGCGKSTTGRALLRLVETQGGSITFNGQRID
TLTGSKLQALRRDIQFIFQDPYASLDPRQTVGYSIMEPLRVHRLLEGDAARERVSWLLERVGLEPEHAWRYPHEFSG
GQRQRICIARALALNPKVVIADESVSALDVSIRAQIINLMLDLQREMGIAFLFISHDMAVVERISHRVAVMYRGRIV
EIGPRRAVFENPQHPYTRKLMAAVPVADPAHRRPQRVLLSDELPGNIFKRGEEGIGTPLQQVGPGHFVAREPAADVL
AR

B

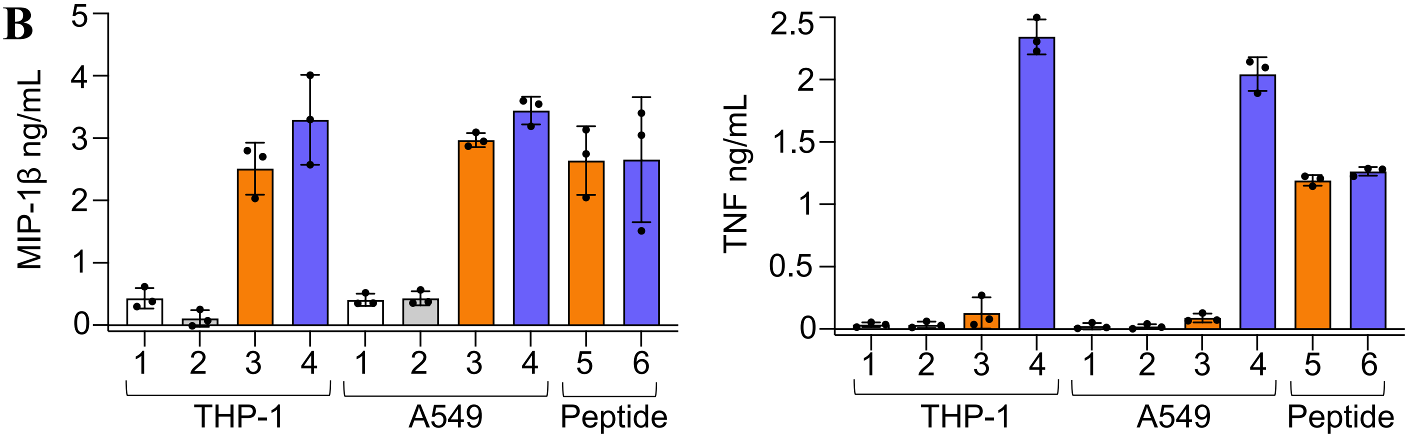

Key: **1.** WT **2.** +*HLA A*24:02* **3.** +*HLA A*24:02* + *fes* **4.** +*HLA A*24:02* + *gsiA*
5. RYPRLFGIV peptide (*fes*) **6.** SLPRLFPLL peptide (*gsiA*)

C

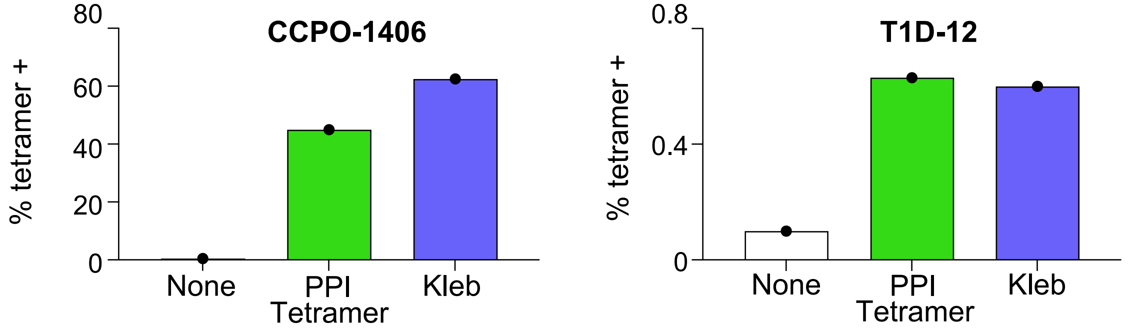

Supplementary Figure 8: Preproinsulin and *Klebsiella oxytoca* crossreactive T-cells are present in multiple patients and the *Klebsiella* derived peptides are genuine epitopes. (**A**) Amino acid sequences of two *Klebsiella oxytoca* proteins with respective epitopes (RYPRLFGIV and SLPRLFPLL) highlighted in color and underlined. Genes (*fes* or *gsiA*) encoding these proteins were used for lentiviral transduction of antigen presenting cells for epitope validation. (**B**) Overnight activation assays with HLA A*24:02 restricted preproinsulin CD8 T-cell clone 4C6 with THP-1 or A549 cell lines co-transduced with *HLA A*24:02* and *fes* or *gsiA* from *Klebsiella oxytoca*. Exogenous RYPRLFGIV and SLPRLFPLL peptides used at 10^{-5} M. Supernatants used for MIP-1β (left) or TNF (right) ELISAs, with error bars depicting the standard deviation of triplicate conditions. MIP-1β or TNF production for T-cell alone conditions have been subtracted from the displayed data. Data using C1R antigen presenting cells shown in **Figure 5**. (**C**) T-cell lines created from HLA A*24^{+} type 1 diabetic patients by enrichment with HLA A*24:02 preproinsulin (PPI, LWMRLLPLL) tetramers were subsequently stained with *Klebsiella* (Kleb)-SLPRLFPLL or PPI tetramers.

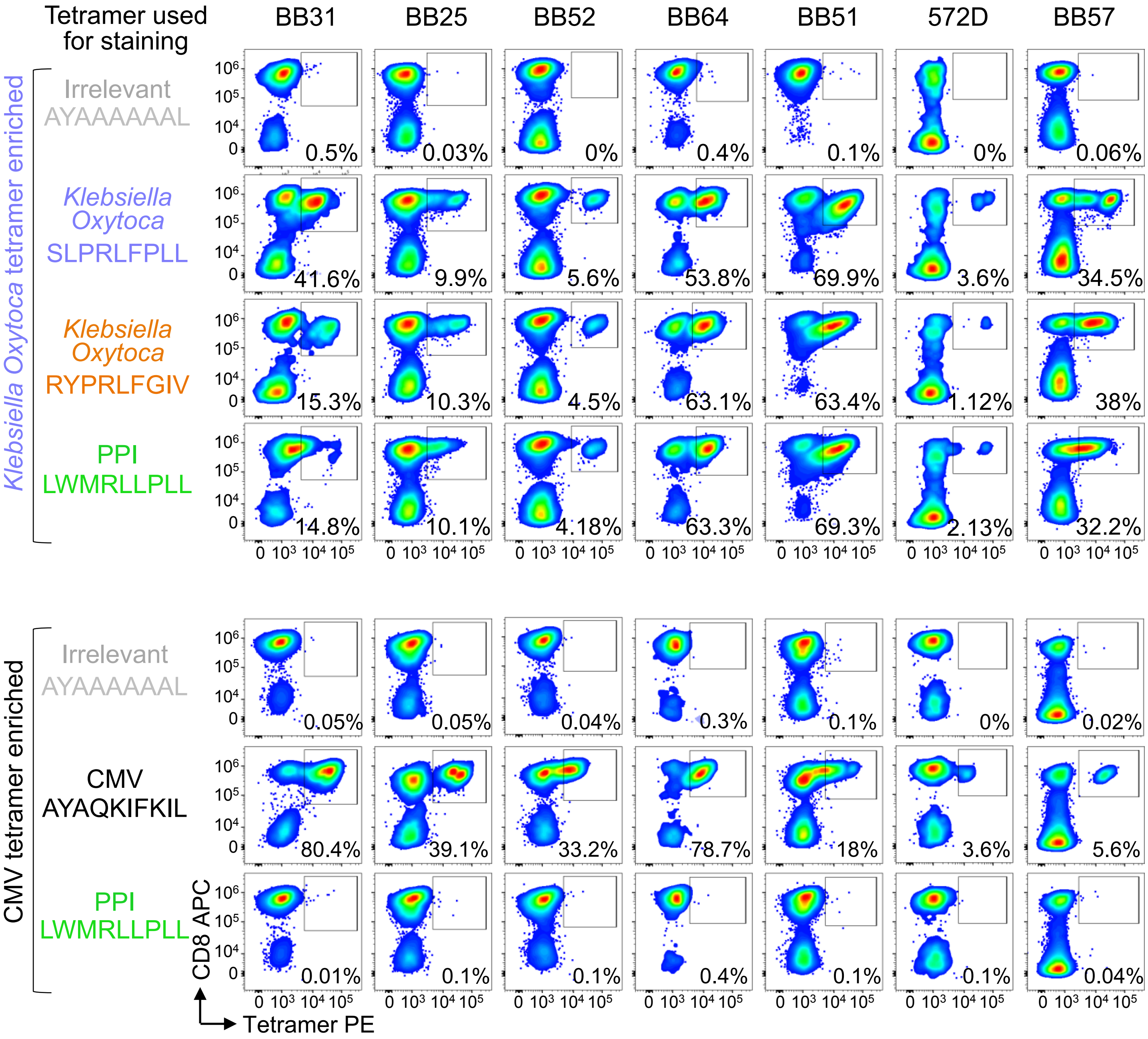

Supplementary figure 9: Klebsiella tetramers co-select preproinsulin specific T-cells in non-diabetic donors. PBMCs from 8 HLA A*24⁺ healthy donors were enriched in parallel with HLA A*24:02 *Klebsiella Oxytoca* (SLPRLFPLL) or CMV (AYAQKIFKIL) PE conjugated tetramers and anti-PE magnetic beads. Following 2 weeks of expansion with allogeneic PBMCs and PHA the T-cell lines were stained with irrelevant (HLA A24 AYAAAAAAL), preproinsulin (PPI) (LWMRLLPLL), *Klebsiella Oxytoca* (SLPRLFPLL and RYPRLFGIV) or CMV (AYAQKIFKIL) tetramers, depending on the initial enrichment. Percentage of tetramer⁺ T-cells is shown.

	Frequency of TCR in tetramer sorted cells: Kleb tetramer	Frequency of TCR in tetramer sorted cells: PPI Tetramer	V-gene	CDR1	CDR3	J-gene
4C6	-	-	TRAV5	DSSSTY	CAEPSGNTGKLIF	TRAJ37*01
	-	-	TRBV7-9	SEHNR	CASSLHHEQYF	TRBJ2-7
BB51	98%	97%	TRAV1-1	TSGFYG	CAVRGGDDKIIF	TRAJ30
	96%	97%	TRBV20-1	DQFATT	CSARDDRGLLRGYNEQFF	TRBJ2-1
BB52	100%	100%	TRAV34	KTLYG	CGADIGDYKLSF	TRAJ20
	96%	100%	TRBV10-3	ENHRY	CAISESVVHTDTQYF	TRBJ2-3
BB57	68%	77%	TRAV38-2	TSESDYY	CAYSHSGNTPLVF	TRAJ29
	20%	18%	TRAV5	DSSSTY	CAVSPAAGNKLTF	TRAJ17
	6.7%	1.5%	TRAV26-1	TISGNEY	CIVRVRGGSSNTGKLIF	TRAJ37*02
	2.9%	2.9%	TRAV12-2	DRGSQS	CAVNSGGFGNVLHC	TRAJ35
	55%	66%	TRBV9	SGDLS	CASSPGQGYQETQYF	TRBJ2-5
	25%	22%	TRBV9	SGDLS	CASSVVGTSGPYLTDTQYF	TRBJ2-3
	8.4%	4.5%	TRBV5-4	SGHNT	CASSYIGGDEQYF	TRBJ2-7
	4.7%	5.7%	TRBV6-1	MNHNS	CASSPGTYLWETQYF	TRBJ2-5
BB64	96%	95%	TRAV20	VSGLRG	CAVGPPYGGSQGNLIF	TRAJ42
	84%	80%	TRBV27	MNHEY	CASSSSPLASAMEQYF	TRBJ2-7
	7.9%	9.8%	TRBV6-5	MNHEY	CASSYTGWGSKETQYF	TRBJ2-5
	3.6%	4.4%	TRBV4-1	MGHRA	CASSQDGETQYF	TRBJ2-5
	1.8%	2.9%	TRBV5-6	SGHDT	CASSLVRSNYGYTF	TRBJ1-2
	1.2%	1.9%	TRBV20-1	DFQATT	CSARDTTGTSGAETQYF	TRBJ2-5

Key: TCR α chains | TCR β chains | non-germline and diversity gene regions (underlined)

α chain similarities | Main 4C6 TCR contacts with PPI peptide

Supplementary Figure 10: Crossreactive T-cell receptors recognizing *Klebsiella* and preproinsulin bear CDR1 and CDR3 similarities to the 4C6 TCR. T-cell lines from healthy HLA A*24^{+} donors, by enrichment detailed in (A), were subsequently sorted by flow cytometry with on Kleb or PPI tetramers and next generation sequencing performed for TCR clonotyping. TCRs shared between the Kleb and PPI sorted T-cells are shown, with their frequency from each tetramer sorted population shown on the left. The TCR sequence of the 4C6 clone is included for comparison, with residues that make contacts with the PPI peptide highlighted with colored circles. Alpha TCR chain similarities to the 4C6 TCR and between TCRs from the donors are colored according to the key. The specific allele for TRAJ37 is indicated, as *01 and *02 encode for a Glycine or Serine respectively at the same position (G-S-G/S-N-T-G-K-L-I-F).

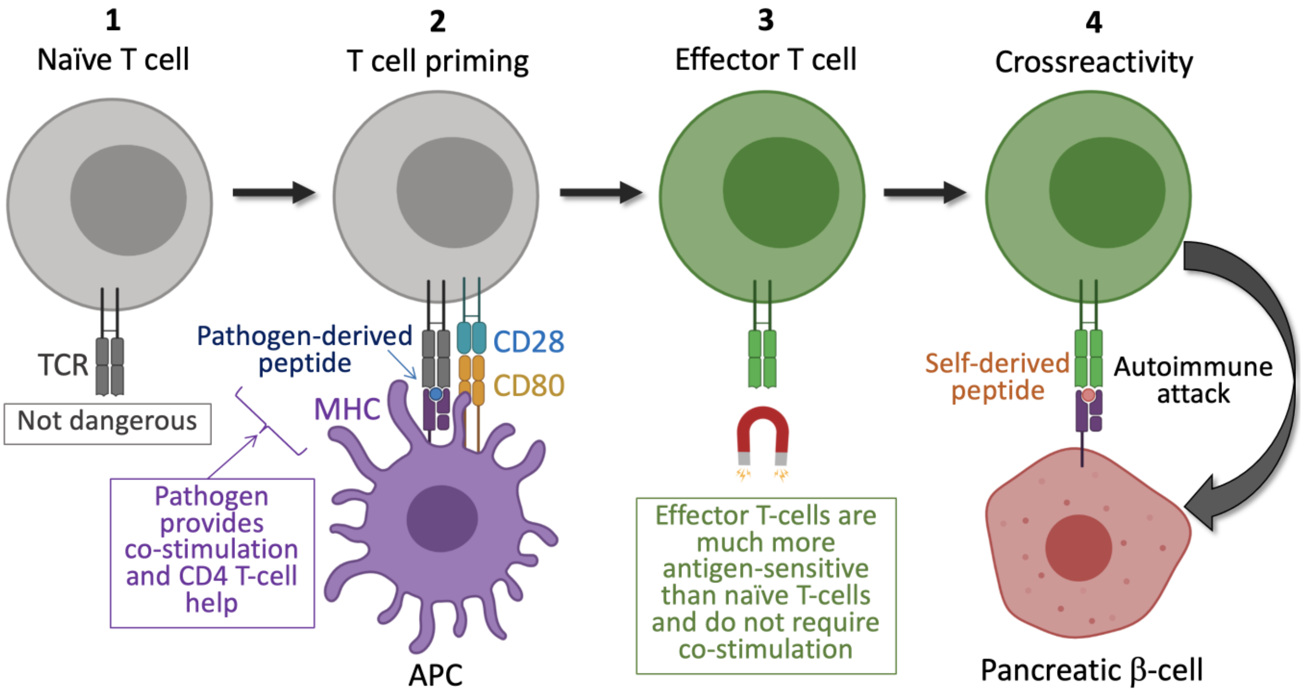

Supplementary figure 11: Induction of type 1 diabetes by molecular mimicry. (**1**) TCRs on naïve T-cells can bind weakly to diabetogenic epitopes presented by disease-risk HLA-I molecules. These T-cells are not dangerous. (**2**) Rarely, T-cells with capacity to weakly bind to diabetogenic epitopes are primed by interaction with a pathogen-derived epitope. This activation requires co-signalling by accessory molecules such as CD80 only found on professional antigen presenting cells (APC) like dendritic cells and help from pathogen-specific CD4 T-cells. (**3**) Activation produces an effector T-cell which is >100-fold more sensitive to TCR stimulation. (**4**) If peripheral tolerance mechanisms fail, such antigen-experienced T-cells have capacity to attack insulin-producing pancreatic β-cells and induce type 1 diabetes.

SUPPLEMENTAL MATERIAL

Supplemental Acknowledgements

TIRID (T-cell Immune Response In Diabetes) Consortium. List of Study Investigators

Investigator	Institution	Location
Professor John Geen	Cwm Taf Morgannwg University health board	Wales, UK
Dr Rebekah Pryce	Cardiff and the Vale University health board	Wales, UK
Dr Ambika Shetty	Cardiff and the Vale University health board	Wales, UK
Dr Onyebuchi Okosieme	Cwm Taf Morgannwg University health board	Wales, UK
Dr Karen Logan	Imperial College Healthcare NHS Trust	London, UK
Dr Ceri Lynch	Cwm Taf Morgannwg University health board	Wales, UK
Dr Alan Dodd	Cwm Taf Morgannwg University health board	Wales, UK

Supplementary Table 1: X-ray Crystallography statistics for presented 3D structures

**PDB Entry**	**7NMD**	**7NME**	**7NMF**	**7NMG**	
**Epitope**	HLA A*24:02-QLPRLFPLL	4C6-HLA A*24:02-QLPRLFPLL Orth	4C6-HLA A*24:02-QLPRLFPLL Mono	4C6-HLA A*24:02-LWMRLLPLL	
**Data Collection**					
Diamond Beamline	DLS-I03	DLS-I03	DLS-I03	DLS-I04	
Date	2019-01-17	2015-07-22	2015-07-22	2016-01-21	
Wavelength	0.97625	0.97623	0.97623	0.97949	
**Crystal Data**					
Crystallisation Conditions	20% PEG 4000, 0.1 M TRIS, pH 7.5, 15% glycerol	25% PEG 1500, 0.1M PCTP Buffer, pH 9.0	20% PEG 3350, 0.2M NaBr, 0.1 M Bis-Tris Propane, pH 7.5	25% PEG 1500, None 0.1M PCTP Buffer, pH 9.0	9.0
a,b,c (Å)	79.54, 48.68, 123.07	55.16, 71.35, 220.18	63.38, 72.25, 114.84	54.32, 72.13, 230.65	
α,β,γ (°)	90.0, 105.43, 90.0	90.0, 90.0, 90.0	90.0, 102.98, 90.0	90.0, 90.0, 90.0	
Space group	$P\ 1\ 2_1\ 1$	$P\ 2_1\ 2_1\ 2_1$	$P\ 1\ 2_1\ 1$	$P\ 2_1\ 2_1\ 2_1$	
Resolution (Å)	2.25 – 73.99	2.20-110.09	2.98 – 60.08	2.48 – 52.93	
Outer shell	2.25 – 2.31	2.20-2.27	2.98 – 3.06	2.48 – 2.58	
R-merge (%)	14.9 (121.7)	16.3 (136.4)	13.1 (69.1)	8.4 (117.5)	
R-pim	8.9 (74.1)	8.7 (76.2)	8.8 (46.8)	5.0 (69.0)	
R-meas (%)	11.9 (93.8)	20.0 (173.1)	15.8 (83.8)	10.8 (148.6)	
CC1/2	0.984 (0.400)	0.989 (0.355)	0.987 (0.644)	0.997 (0.513)	
I / σ(I)	5.4 (1.0)	5.9 (1.3)	7.8 (1.7)	11.6 (1.3)	
Completeness (%)	99.0 (98.3)	99.5 (95.1)	96.6 (98.1)	99.8 (99.9)	
Multiplicity	3.7 (3.6)	5.1 (5.2)	2.8 (2.9)	4.4 (4.4)	
Total Measurements	160,482 (11,406)	230,237 (20,094)	57,388 (9,519)	144,009 (16,321)	
Unique Reflections	43,149 (3,179)	44,950 (3,864)	20,167 (3,295)	33,057 (3,692)	
Wilson B-factor($Å^2$)	37.2	30.9	56.8	42.3	
**Refinement Statistics**					
Resolution Range Used	2.25 – 74.01	2.20 – 110.09	2.98 – 60.08	2.48 – 52.93	
Non-H Atoms	6,657	6,814	6,682	6,763	
R-work reflections	41,059	42,709	19,178	31,375	
R-free reflections	2,050	2,169	1,426	1,616	
R-work/R-free (%)	20.9 / 26.5	21.5 / 27.4	18.2 / 25.9	19.8 / 27.1	
**rms deviations (target)**					
Bond lengths (Å)	0.012 (0.013)	0.007 (0.013)	0.012 (0.013)	0.011 (0.013)	
Bond Angles (°)	1.496 (1.660)	1.168 (1.654)	1.570 (1.652)	1.452 (1.653)	
[1]Coordinate error	0.244	0.219	0.413	0.290	
Mean B value ($Å^2$)	47.6	38.1	61.2	51.8	
**Ramachandran Statistics**					
Favoured/allowed/Outliers	735 / 20 / 3	774 / 26 / 6	776 / 28 / 5	757 / 46 / 4	
%	97.0 / 2.6 / 0.4	96.0 / 3.2 / 0.7	95.9 / 3.5 / 0.6	93.8 / 5.7 / 0.5	

* One crystal was used for determining each structure.

* Figures in brackets refer to outer resolution shell, where applicable.

1 Coordinate Estimated Standard Uncertainty in (Å), calculated based on maximum likelihood statistics

Supplementary Table 2: Molecular contacts in the 4C6-HLA A*24:02-LWMRLLPLL cocrystal structure

CDR loop	TCR residue	Peptide residue	MHC residue	VdWs (≤4 Å)	H-bonds (≤3.4 Å)	Salt bridges
CDR1α	Asp27	Arg4		1	1	
	Ser29	Arg4		3	1	
	Ser29		Thr163	3		
	Ser30	Arg4		4	2	
	Thr31		Gln155	4	1	
	Thr31		Ala158	3		
	Thr31		Tyr159	3		
	Tyr32	Leu5		1		
	Tyr32		Gln155	1		
CDR2α	Phe51		Glu154	3		
	Phe51		Gln155	5		
	Ser52		Ala158	1		
	Asn53		Arg157	2		
	Asn53		Ala158	1		
	Asn53		Glu161	3	1	
FWα	Lys68		Gly162	2		
	Lys68		Asp166	1		
CDR3α	Pro93	Arg4		2	1	
	Ser94	Arg4		3		
	Gly95	Arg4		5	3	
	Asn96	Arg4		6	1	
	Asn96		Gly65	3		
	Asn96		Lys66	6	1	
	Asn96		Ala69	2		
	Thr97	Arg4		1		
	Thr97	Leu5		3		
	Gly98	Leu5		1		
CDR1β	Asn31	Leu8		3		
	Asn31		Lys146	1	1	
	Arg32	Leu5		1		
	Arg32	Leu6		3	1	
CDR2β	Gln51	Leu8		5		
	Gln51		Gln72	1		
	Gln51		Thr73	4	2	
	Asn52		Glu76	4	1	
	Glu53		Arg79	1	1	
	Leu56		Gln72	2		
CDR3β	Leu97		Lys146	1		
	Leu97		Ala150	1		
	His98	Leu5		7		
	His98	Leu6		2		
	His98	Pro7		5		
	His98		Val152	3		
	His98		Gln155	2	1	
	His99	Leu5		1		
	His99		Ala150	1	1	
	His99		His151		1	
	His99		Gln155	2		
	Glu100		His151	2		

Contact summary: 147 total (67 between peptide and 4C6 TCR; 34 with Arg4 and 14 with Leu5)

Supplementary Table 3: Molecular contacts in the 4C6-HLA A*24:02- QLPRLFPLL cocrystal structure

CDR loop	TCR residue	Peptide residue	MHC residue	VdWs (≤4 Å)	H-bonds (≤3.4 Å)	Salt bridges
CDR1α	Ser29	Arg4		4	1	
	Ser29		Thr163	2		
	Ser30	Arg4		4	2	
	Thr31		Gln155	3	1	
	Thr31		Ala158	2		
	Thr31		Tyr159	2		
CDR2α	Phe51		Glu154	2		
	Phe51		Gln155	3		
	Ser52		Ala158	2		
	Asn53		Ala158	1		
	Asn53		Glu161	3	1	
FWα	Lys68		Glu162	1		
CDR3α	Pro93	Arg4		4	1	
	Ser94	Arg4		3		
	Gly95	Arg4		4	2	
	Asn96	Arg4		6	1	
	Asn96		Gly65	2		
	Asn96		Lys66	4		
	Asn96		Ala69	1		
	Thr97	Leu5		2		
	Gly98	Leu5		2		
CDR1β	Glu29		Lys146	1		
	Asn31	Leu8		1		
	Asn31		Lys146	2		
	Arg32	Phe6		2	1	
	Arg32	Leu8		2		
	Arg32		Thr73	1		
CDR2β	Gln51	Leu8		2		
	Gln51		Gln72	3		
	Gln51		Thr73	4	1	
	Asn52		Glu76	6	1	
	Leu56		Gln72	3		
CDR3β	Leu97		Ala150	1		
	His98	Leu5		3		
	His98	Phe6		2		
	His98	Pro7		5		
	His98		Val152	3		
	His98		Gln155	3	1	
	His99		Ala150	1	1	
	His99		His151	1		
	Glu100		His151		1	

Contact summary: 118 total (52 between peptide and 4C6 TCR; 32 with Arg4 and 7 with Leu5)
